# Distinct classes of gut bacterial molybdenum-dependent enzymes produce urolithins

**DOI:** 10.1073/pnas.2501312122

**Published:** 2025-12-24

**Authors:** Minwoo Bae, Xueyang Dong, Julian Avila-Pacheco, Quyen D. Nguyen, Fechi Inyama, Vayu Hill-Maini, Clary B. Clish, Emily P. Balskus

**Affiliations:** ^a^Department of Chemistry and Chemical Biology, Harvard University, Cambridge, MA 02138; ^b^Broad Institute of Massachusetts Institute of Technology and Harvard, Cambridge, MA 02142; ^c^HHMI, Harvard University, Cambridge, MA 02138

**Keywords:** molybdenum enzyme, gut microbiome, diet, polyphenol, urolithin

## Abstract

Urolithin A is an anti-aging and anti-inflammatory gut bacterial metabolite derived from ellagic acid (EA), a polyphenol abundant in berries and nuts. The conversion of EA to urolithin A involves multiple chemically challenging phenol dehydroxylation steps that produce urolithins with varying bioactivities. Despite their biological and chemical significance, the bacterial enzymes responsible for urolithin production remain largely unidentified. Here, we use differential gene expression analysis, anaerobic protein production, and enzyme assays to identify members of two distinct molybdenum enzyme families (the DMSO reductase family and the xanthine oxidase family) capable of regioselective dehydroxylation and urolithin generation. These two enzyme families have distinct substrate requirements, suggesting they employ different catalytic mechanisms for phenol dehydroxylation. Multiomics analysis of a human cohort uncovers decreased levels of urolithin A and genes encoding urolithin A-producing enzymes in patients with inflammatory bowel disease (IBD), implying reduced health effects of EA consumption in this setting. Together, this study elucidates the molecular basis of urolithin production, expands the known enzymatic repertoire of the human gut microbiome, and suggests a potential link between gut bacterial urolithin production and reduced host inflammation.

## Significance

The human gut microbiome modulates the health effects of dietary compounds by modifying their chemical structures. Gut microbes extensively metabolize polyphenols, a group of diverse plant-derived compounds associated with positive health outcomes. Urolithin A, a gut bacterial metabolite derived from a polyphenol abundant in berries and nuts, exhibits potent anti-inflammatory activity. However, the gut bacterial enzymes involved in its production remain largely unknown. Here, we report urolithin-producing gut bacterial enzymes, including four phenol dehydroxylases from two distinct molybdenum-dependent enzyme families. Analyzing human gut microbiomes suggests a potential link between these genes and reduced inflammation. Together, our findings map urolithin production to enzymes, increasing our understanding of how the gut microbiome can alter the impacts of diet on human health.

**Fig. 1.**
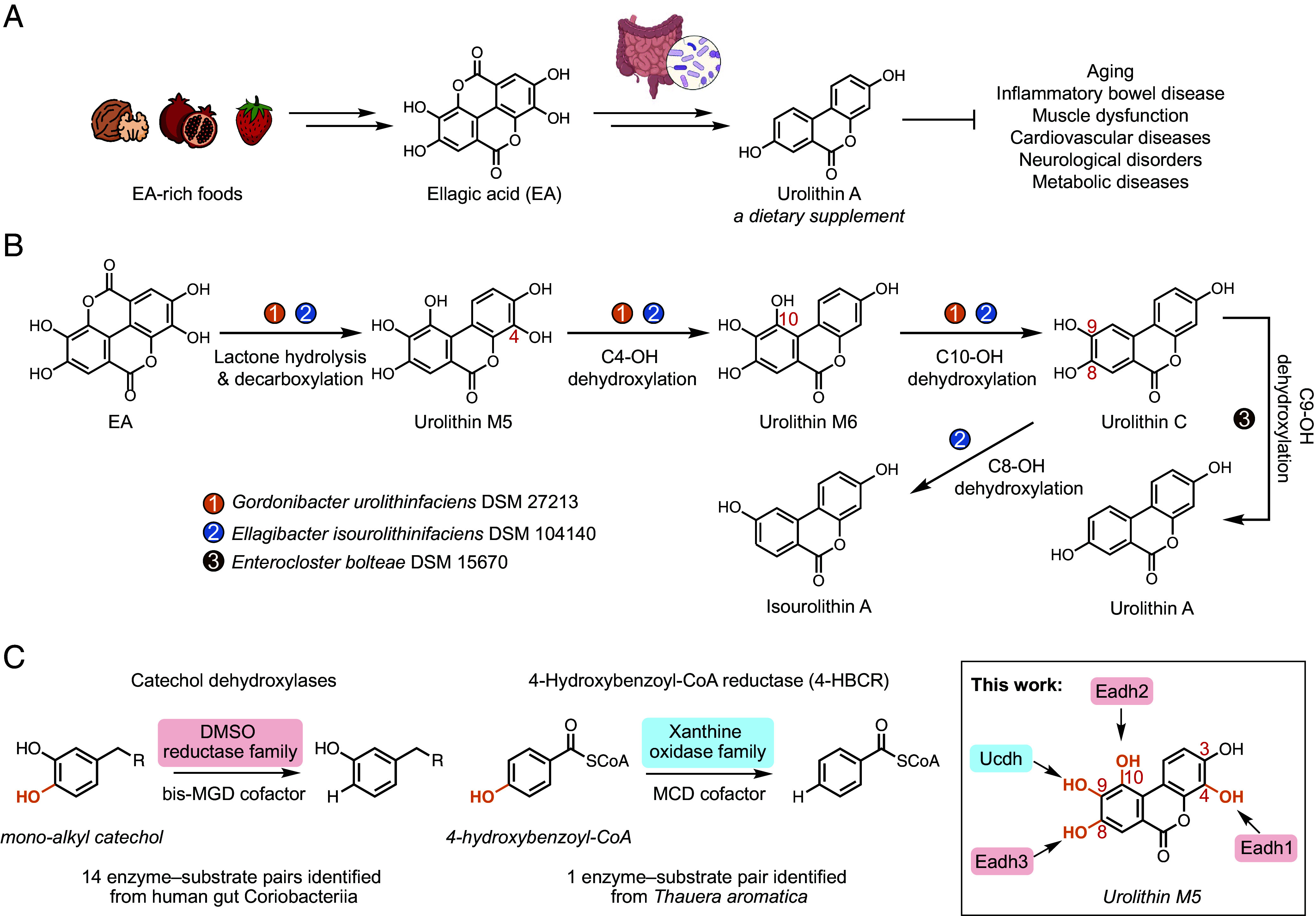
Unknown gut bacterial enzymes produce urolithin A. (*A*) The beneficial health effects of urolithin A are derived from gut microbial metabolism of the dietary polyphenol ellagic acid (EA). (*B*) Major metabolic pathways for EA by previously reported gut bacterial species. The pathways involve urolithins with different hydroxylation patterns. (*C*) Comparison between phenol dehydroxylations performed by known enzymes (catechol dehydroxylases and 4-HBCR) and dehydroxylations involved in EA metabolism. Phenol groups of urolithin M5 subject to dehydroxylation are highlighted in orange.

EA is a polyphenol derived from ellagitannins abundant in foods such as berries, nuts, and pomegranates (1). Consumption of EA-rich foods is associated with positive health outcomes such as improved cardiovascular health and decreased mortality (2–5). These health benefits have partly been attributed to the urolithins, a group of EA-derived metabolites produced by gut bacteria (6). Urolithin A, the urolithin whose bioactivity has been most extensively studied, inhibits proinflammatory cytokine production and restores mitophagy in aged cells (7–11). In model organisms, the activities of urolithin A have been linked to reduced inflammation (12–14), activation of antitumor immunity (15), and protection against age-related conditions such as repressed stem cell function (16), muscle dysfunctions (7, 8, 17), neurological disorders (18–20), and metabolic diseases (21–23) (Fig. 1*A*). Recent clinical studies demonstrated that urolithin A administration improved muscle function in elderly and middle aged human subjects (24–26), and urolithin A is currently marketed as a dietary supplement. Importantly, fecal urolithin A levels vary widely across individuals even when EA consumption is controlled, presumably due to varied gut microbial metabolic capabilities (27–29). Despite the biological importance of gut bacterial urolithin A production, only few genes and enzymes involved in this metabolism have been uncovered.

Intriguingly, the conversion of EA to urolithin A involves a series of phenol dehydroxylations that can yield urolithins with different hydroxylation patterns. Recent studies have shown that the human gut Coriobacteriia species *Gordonibacter urolithinfaciens* converts EA to urolithin C, which is further metabolized to urolithin A by *Enterocloster bolteae*, a gut Clostridial species (Fig. 1*B*) (30, 31). These two species remove three hydroxyl groups of urolithin M5 at C4, C10, and C9 to produce urolithin M6, urolithin C, and urolithin A in sequence. Alternatively, the gut Coriobacteriia species *Ellagibacter isourolithinifaciens* produces a regioisomer of urolithin A called isourolithin A via dehydroxylation of urolithin M5 at C4, C10, and C8 (Fig. 1*B*) (32). Importantly, despite the subtle changes in their structures, urolithins with different hydroxylation patterns exhibit different bioactivity in vitro (33–36). For example, urolithin A, but not isourolithin A, significantly reduced triglyceride accumulation in human adipocytes (33). Dietary intervention studies have revealed that individuals who metabolize EA to urolithin A have improved health outcomes, including lower rates of obesity and lower levels of cardiovascular disease biomarkers compared to a group of people who produce isourolithin A and its congener urolithin B (37–39). Therefore, the regioselectivity of urolithin dehydroxylation may affect the health consequence of EA consumption.

Reductive phenol dehydroxylation is a transformation of high chemical interest as it is a key reaction in lignin valorization (40, 41). However, current methods are energy-intensive, requiring high temperature and high H_2_ partial pressure to activate the stable aromatic C–O bond (42). Interestingly, this transformation is central to anaerobic bacterial metabolism of aromatic compounds and has been linked to two types of molybdenum-dependent enzymes (Fig. 1*C*). Notably, catechol dehydroxylases, members of the dimethyl sulfoxide (DMSO) reductase family, catalyze the dehydroxylation of a catechol motif (i.e., 1,2-dihydroxylated benzene) using a bis-molybdopterin guanidine dinucleotide (bis-MGD) cofactor (43–47). Primarily encoded in the genomes of gut Coriobacteriia, characterized catechol dehydroxylases process polyphenols with high substrate specificity (43–45, 48, 49) and have been shown to support anaerobic respiration (45, 48, 50). Another phenol dehydroxylase, 4-hydroxybenzoyl-CoA reductase (4-HBCR), removes the hydroxyl group of 4-hydroxybenzoyl-CoA, a key step in anaerobic catabolism of aromatic compounds (51). Found in the sewage-derived bacterium *Thauera aromatica* (52), 4-HBCR is a member of the xanthine oxidase family and carries a molybdenum cytosine dinucleotide (MCD) cofactor (53). A recent study independently linked a xanthine oxidase family enzyme in *E. bolteae* to the dehydroxylation of urolithin C to urolithin A (54). However, the biochemical function of this protein has not been demonstrated in vitro, and the identities of the enzymes responsible for urolithin metabolism in gut Coriobacteriia species remain unknown. The serial dehydroxylations observed in EA bioactivation, which occur on a highly oxygenated aromatic scaffold, offer an exciting opportunity for further discovery of phenol dehydroxylases.

Here, we elucidate the gut bacterial enzymes involved in the conversion of EA to urolithin A and the competing pathways to isourolithin A and urolithin B. Using differential gene expression analysis, protein expression, and enzyme activity assays, we identify four urolithin dehydroxylases: three catechol dehydroxylases (Eadh1, Eadh2, and Eadh3) from *Gordonibacter* strains and *E. isourolithinifaciens* and a xanthine oxidase family enzyme (Ucdh) from *E. bolteae* (Fig. 1*C*). By examining substrate scope and enzyme kinetics, we reveal that these enzymes target individual hydroxyl groups on the urolithins and exhibit substrate specificity that explains the sequence of this metabolic pathway. Finally, we find that both Coriobacteriia EA-metabolizing gene abundance and urolithin A levels are depleted in IBD patients, suggesting a potential route through which inflammation alters production of the bioactive metabolite urolithin A. Altogether, this work elucidates the molecular basis of gut bacterial urolithin production and lays the groundwork for future efforts to understand polyphenol dehydroxylation and its relevance for human health.

## Results

### Identification of a Coriobacteriia Gene Cluster Responsible for EA Metabolism.

We hypothesized that *Gordonibacter* and *Ellagibacter* species likely employ catechol dehydroxylases for urolithin metabolism, given that strains of these species typically encode over 30 uncharacterized members of this enzyme family (48). Since catechol dehydroxylases are also widespread across other gut Coriobacteriia, we screened a library of Coriobacteriia strains for EA metabolism in liquid culture using LC–MS/MS quantification. Consistent with previous findings (30, 32), urolithin C was produced by *G. urolithinfaciens* and *Gordonibacter pamelaeae*, while isourolithin A was generated by *E. isourolithinifaciens* (*SI Appendix*, Fig. S1*A*). In cultures of *G. urolithinfaciens* and *G. pamelaeae*, the intermediates urolithin M5 and urolithin M6 were also detected. Additionally, we found another human gut *Gordonibacter* strain, *Gordonibacter* sp. (*Gs*) 28C, completely converted EA to urolithin C. All other Coriobacteriia strains we tested were inactive (only EA was recovered), and none of the strains produced urolithin A. We then investigated whether this metabolic activity is induced by EA, as transcription of genes encoding catechol dehydroxylases is typically induced by their cognate substrates (43–45, 55). The cell lysate of *Gs* 28C converted EA to urolithin C when the culture was grown with EA, whereas lysate from vehicle-treated culture showed no activity (*SI Appendix*, Fig. S1*B*). This result indicated that the enzymes responsible for EA metabolism are expressed in response to the substrate, similar to other catechol dehydroxylases.

**Fig. 2.**
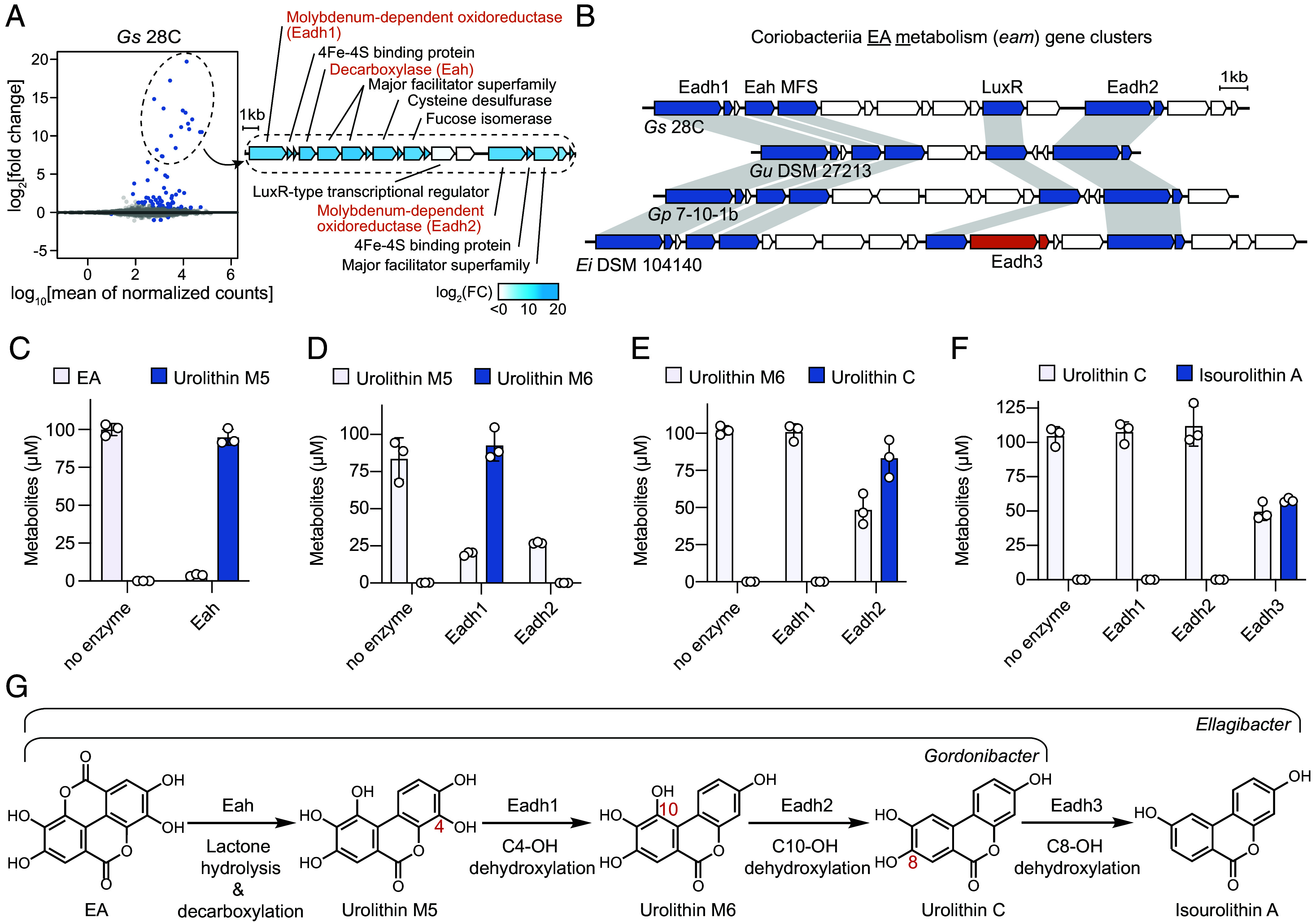
Identification of a Coriobacteriia gene cluster responsible for EA metabolism. (*A*, *Left*) Differential gene expression analysis of *Gs* 28C cultured in media supplemented with 100 µM EA or vehicle (n = 3 biological replicates). Genes with adjusted *P* value < 0.01 are represented by navy dots. (*Right*) Functional annotations for proteins encoded by the gene cluster induced by EA. (*B*) Conservation of the EA metabolism (*eam*) gene cluster among EA-metabolizing Coriobacteriia strains. The conserved genes are colored navy. Genes encoding an additional catechol dehydroxylase homolog (Eadh3) in *E. isourolithinifaciens* are colored orange. (*C*) Transformation of EA to urolithin M5 by Eah. 1 µM Eah was incubated with 100 µM EA at rt for 20 h in a pH 7.0 buffer (50 mM HEPES and 250 mM NaCl) under anaerobic conditions. (*D*) Metabolism of urolithin M5 by Eadh1 and Eadh2. (*E*) Metabolism of urolithin M6 by Eadh1 and Eadh2. (*F*) Transformation of urolithin C by Eadh1, Eadh2, and Eadh3. (*D*–*F*) 100 nM enzyme was incubated with 100 µM substrate, 200 µM methyl viologen, and 100 µM sodium dithionite at rt for 20 h in a pH 7.0 buffer (20 mM MOPS and 300 mM NaCl) under anaerobic conditions. (*C*–*F*) Data represented as mean ± SD with n = 3 replicates. (*G*) Assignment of enzymes in the metabolism of EA by *Gordonibacter* and *Ellagibacter* strains. Gs, Gordonibacter species; Gu, Gordonibacter urolithinfaciens; Gp, Gordonibacter pamelaeae; Ei, Ellagibacter isourolithinifaciens; MFS, major facilitator superfamily.

To identify putative EA-metabolizing genes, we performed a differential gene expression analysis of *Gs* 28C treated with either EA or vehicle, a strategy that has been previously used to link catechol substrates to their dehydroxylases (43–45). We identified 13 genes that were upregulated by more than 1,000-fold in response to EA compared to vehicle (Fig. 2*A*). These genes mapped onto a single gene cluster that encoded two putative molybdopterin-dependent oxidoreductases (Eadh1 and Eadh2) and additional proteins related to EA metabolism. A protein phylogenetic analysis placed these two enzymes in the catechol dehydroxylase clade alongside known catechol dehydroxylases from *Gordonibacter* strains (*SI Appendix*, Fig. S1*C*). Like other *Gordonibacter*-type catechol dehydroxylases (45), the genes encoding Eadh1 and Eadh2 are colocalized with genes encoding 4Fe-4S binding proteins (Fig. 2*A*). Considering that EA metabolism by *Gs* 28C involves two dehydroxylation steps, we reasoned that Eadh1 and Eadh2 may each catalyze one step. By performing a sequence-based homology search with Eadh1 and Eadh2 as queries using Basic Local Alignment Search Tool (BLAST) (56), we identified homologous gene clusters in all known EA metabolizers as well as as-yet-uncultivated Coriobacteriia from human and other animal gut microbiomes (Fig. 2*B* and *SI Appendix*, Fig. S1*D*). Interestingly, the gene cluster from *E. isourolithinifaciens*, which metabolizes EA to isourolithin A using one additional dehydroxylation step (32), encoded an additional uncharacterized molybdopterin-dependent oxidoreductase (Eadh3) that is also found within the catechol dehydroxylase clade (Fig. 2*B* and *SI Appendix*, Fig. S1*C*). We hypothesized that this enzyme processes urolithin C to isourolithin A.

In addition to the putative catechol dehydroxylases, the identified gene clusters all contained genes encoding a decarboxylase (Eah), MFS transporters, and a LuxR-type transcriptional regulator. MFS transporters and LuxR-type transcriptional regulators are often found in the genomic neighborhood of catechol dehydroxylases. The upregulated catechol dehydroxylase homologs (Eadh1/2/3) appear to lack signal peptides like other characterized catechol dehydroxylases from *Gordonibacter* species (45, 46), suggesting that they are cytoplasmic. For these cytoplasmic catechol dehydroxylases, the colocalized MFS transporters are thought to transport substrates into the cell (45). Indeed, the urolithins produced by Coriobacteriia strains were detected in the supernatants of liquid cultures incubated with EA (*SI Appendix*, Fig. S1 *A* and *E*), suggesting that these metabolites are dynamically imported and exported from cells, making them available for metabolism by other species like *E. bolteae.* On the other hand, LuxR-type regulators have been shown to regulate transcription of genes encoding other catechol dehydroxylases (55). Annotated as a metal-dependent decarboxylase (IPR032465) by InterPro 98.0 (57), Eah is a candidate for the initial lactone ring opening and decarboxylation of EA to urolithin M5. We heterologously expressed Eah from *Gs* 28C in *Escherichia coli* BL21 (DE3) and confirmed that the purified protein converted EA to urolithin M5 (Fig. 2*C* and *SI Appendix*, Fig. S1*F*). This suggested that the gene cluster identified by differential gene expression analysis was likely responsible for EA metabolism, and we named this gene cluster as the Coriobacteriia EA metabolism (*eam*) gene cluster.

Next, we sought to characterize the catechol dehydroxylase homologs (Eadh1/2/3) associated with this gene cluster. Using a recently developed cumate-inducible expression system for catechol dehydroxylases (55), we anaerobically expressed and purified recombinant Eadh1 and Eadh2 from *G. urolithinfaciens,* their native host (*SI Appendix*, Fig. S1*G*). Each purified enzyme was incubated with either urolithin M5 or urolithin M6 along with methyl viologen and sodium dithionite under anaerobic conditions, and urolithin substrates and products were quantified by LC–MS/MS using authentic standards for comparison. Consistent with our hypothesis, distinct dehydroxylation steps were catalyzed by Eadh1 (C4-OH dehydroxylation of urolithin M5 to urolithin M6) (Fig. 2*D*) and Eadh2 (C10-OH dehydroxylation of urolithin M6 to urolithin C) (Fig. 2*E*). Similarly, Eadh3 from *E. isourolithinifaciens* was expressed in *G. urolithinfaciens* and purified for enzyme assays (*SI Appendix*, Fig. S1*G*). As predicted, Eadh3 converted urolithin C to isourolithin A via C8-OH dehydroxylation (Fig. 2*F*). Altogether, the activities of Eah, Eadh1, Eadh2, and Eadh3 account for the EA metabolic pathway in *Gordonibacter* and *Ellagibacter* strains (Fig. 2*G*).

### Catechol Dehydroxylase Substrate Specificity and Kinetics Explain Urolithin Production.

**Fig. 3.**
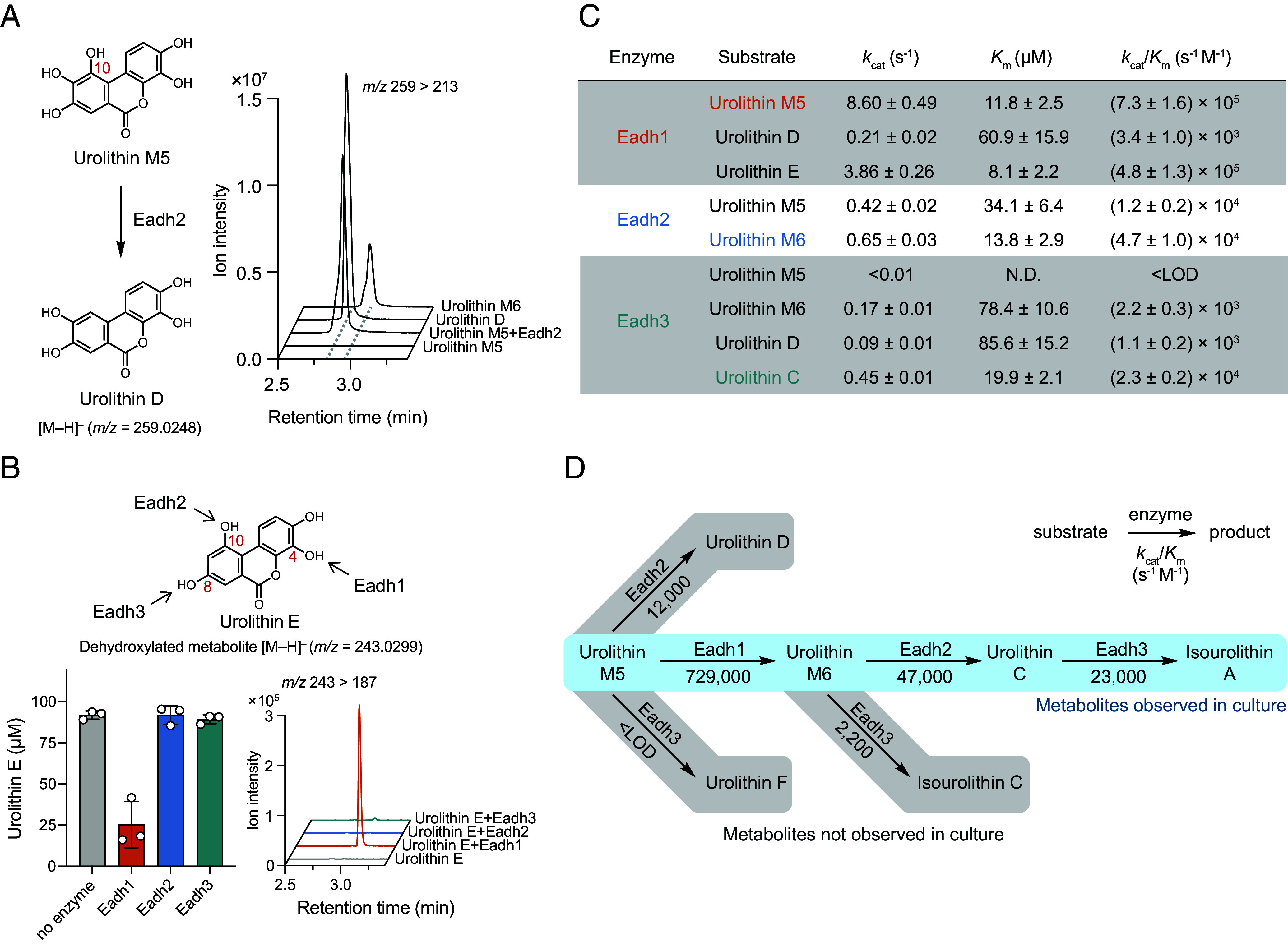
Catechol dehydroxylase substrate specificity and kinetics explain urolithin production. (*A*) LC–MS/MS traces of urolithin M5 metabolism by Eadh2. (*B*) Urolithin E dehydroxylation by Eadh1, Eadh2, and Eadh3 (n = 3). (*A* and *B*) 100 nM enzyme was incubated with 100 µM substrate, 200 µM methyl viologen, and 100 µM sodium dithionite at rt for 20 h in pH 7.0 buffer (20 mM MOPS and 300 mM NaCl) under anaerobic conditions. (*C*) Kinetic parameters of Eadh1, Eadh2, and Eadh3 using initial rates measured in the first 60 s. Data are mean ± SD (n = 3). Fitted parameters are mean ± SE (n = 3) as derived from nonlinear curve fitting to the Michaelis–Menten equation. The reactions were performed in pH 7.0 buffer (20 mM MOPS and 300 mM NaCl) containing varying concentrations of enzymes and substrates, 200 µM methyl viologen, and 100 µM sodium dithionite at 30 °C. (*D*) A scheme of possible metabolic routes for urolithins by Eadh1, Eadh2, and Eadh3 in Coriobacteriia and the catalytic efficiency (*k*_cat_/*K*_m_) of each step. LOD, limit of detection; N.D., not determined.

Surprisingly, we noticed that Eadh2 also consumed urolithin M5 but did not produce urolithin M6 (Fig. 2*D*). Instead, it generated a new mass feature that had the same mass as urolithin M6 but eluted at a different retention time on LC–MS/MS (Fig. 3*A*). Considering that Eadh2 dehydroxylated the C10-OH of urolithin M6, we reasoned this product could be urolithin D, the C10-OH dehydroxylated metabolite of urolithin M5 (58). Indeed, the retention time of the product produced by Eadh2 from urolithin M5 matched that of a urolithin D synthetic standard (Fig. 3*A*). These results showed that Eadh2 dehydroxylates the C10-OH of both urolithin M5 and urolithin M6 substrates, which differ only by a hydroxyl on C4. Likewise, Eadh1, the enzyme removing C4-OH of urolithin M5, can accept urolithin D as a substrate to produce urolithin C via dehydroxylation at C4 (*SI Appendix*, Fig. S2*A*). Eadh3 also demonstrated activity toward multiple C8-OH containing compounds other than urolithin C; however, the identities of the resulting products remain unverified due to a lack of authentic standards (*SI Appendix*, Fig. S2*B*). Importantly, dehydroxylation by these enzymes required the hydroxyl group being removed to be part of a catechol. For example, urolithin E, which has its C4-OH but not its C10 and C8-OHs *ortho* to another hydroxyl group, was accepted as a substrate only by the C4-OH dehydroxylating enzyme Eadh1 (Fig. 3*B*). This requirement is consistent with previous studies of characterized catechol dehydroxylases, which revealed that a catechol is essential for activity (45). Combined, these results suggest that Eadh1, Eadh2, and Eadh3 regioselectively remove catecholic C4, C10, and C8-OHs of urolithins, respectively, with substrate promiscuity in terms of nonparticipating hydroxyl substituents.

The promiscuity of these catechol dehydroxylases implied possible alternative EA metabolic pathways. For example, urolithin M5 could be dehydroxylated in vitro by Eadh1 and Eadh2 to urolithin M6 and urolithin D, respectively. However, urolithin D has not been detected in *Gordonibacter* or *Ellagibacter* liquid cultures incubated with EA, for which urolithin M6 has been proposed as the major intermediate (30, 32). We reasoned that the specific intermediates observed in culture are likely determined by the substrate preferences of Eadh enzymes. To test this hypothesis, we performed enzyme kinetics experiments for each dehydroxylase–substrate pair using a previously reported continuous absorbance-based assay (48). All three enzymes showed varying kinetic parameters toward different urolithin substrates (Fig. 3*C* and *SI Appendix*, Fig. S2*C*). Comparing the kinetic efficiency (*k*_cat_/*K*_m_) for different substrates revealed that urolithin M5, urolithin M6, and urolithin C are the preferred substrates of Eadh1, Eadh2, and Eadh3, respectively. Importantly, these preferences are consistent with the sequence of intermediates observed in culture. For example, the predominant production of urolithin M6 from urolithin M5 by *Gordonibacter* and *Ellagibacter* strains can be attributed to the significantly higher kinetic efficiency of Eadh1 toward urolithin M5 (729,000 s^−1^ M^−1^) compared to the other two enzymes (12,000 s^−1^ M^−1^ by Eadh2 and <limit of detection by Eadh3) (Fig. 3*D*). Similarly, urolithin M6 was metabolized more readily by Eadh2 than by Eadh3, which aligns with the observed formation of urolithin C as a precursor of isourolithin A. Altogether, these enzyme kinetics experiments revealed the native substrates for each dehydroxylase and explain the sequence of EA metabolism observed in culture.

### A Xanthine Oxidase Homolog Catalyzes Dehydroxylation of Urolithin C to Urolithin A.

Next, we sought to identify and characterize the *E. bolteae* enzyme responsible for the C9-OH dehydroxylation of urolithin C to urolithin A. Until recently, phenol dehydroxylations within the human gut have been exclusively attributed to catechol dehydroxylases from Coriobacteriia (43–45, 48, 49). However, *E. bolteae,* a Clostridial species, encodes two DMSO reductase family enzymes (IPR006657) in its genome, both of which share <22% amino acid identity to any characterized catechol dehydroxylases, suggesting that this step may not be catalyzed by this type of enzyme. Notably, unlike the hydroxyl groups removed by characterized catechol dehydroxylases, the C9-OH of urolithin C is positioned *para* to an electron-withdrawing ester group. This electronically resembles 4-hydroxybenzoyl-CoA, the substrate of the xanthine oxidase family enzyme 4-HBCR (Fig. 1*C*), which contains a phenol group *para* to a thioester, a configuration proposed to stabilize catalytic intermediates (59, 60). This analysis suggested that a protein related to 4-HBCR may be involved in the C9-OH dehydroxylation. Indeed, a recent study linked this activity to a xanthine oxidase family enzyme in *E. bolteae* (54).

**Fig. 4.**
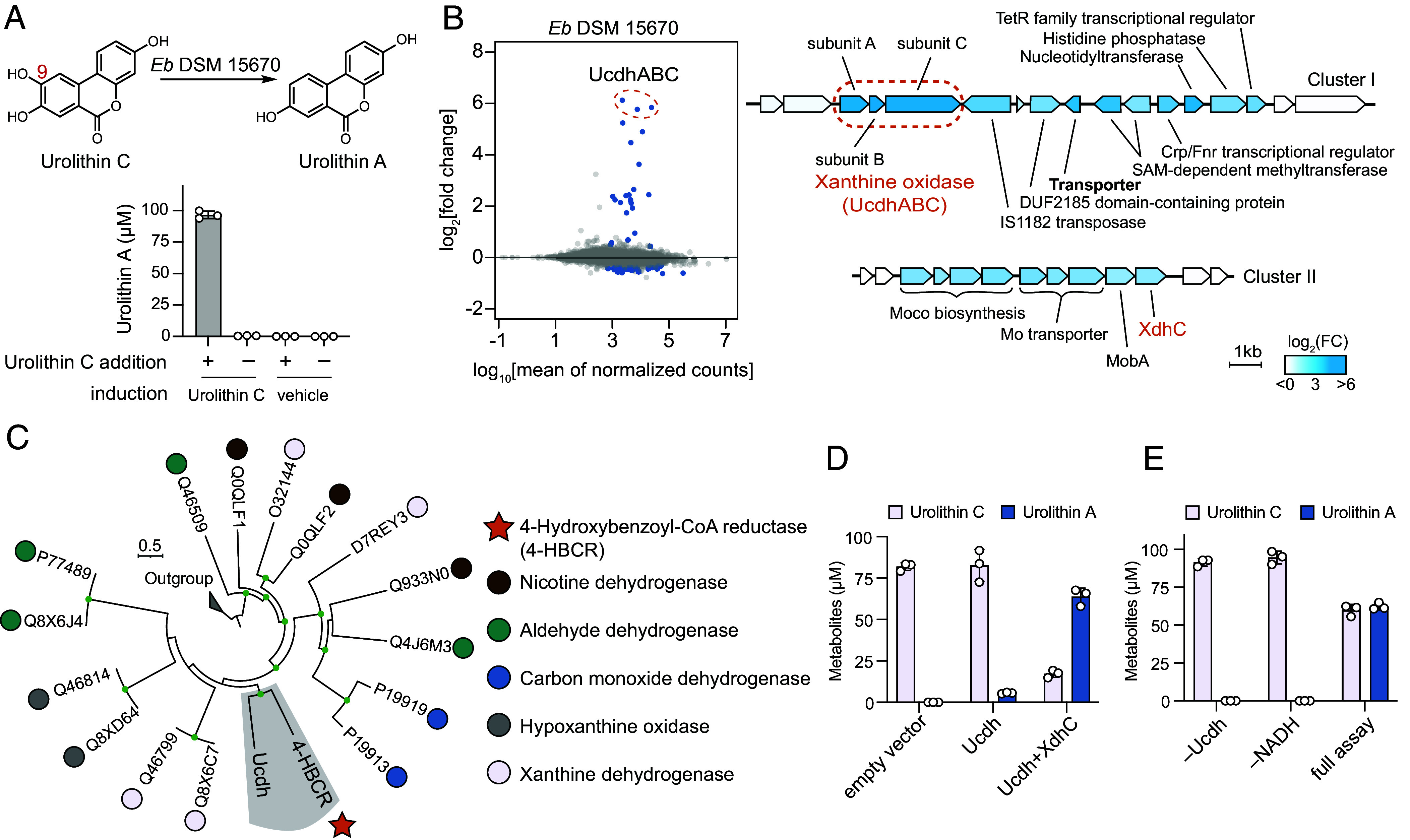
A xanthine oxidase homolog catalyzes dehydroxylation of urolithin C to urolithin A. (*A*) Cell suspensions of *Eb* DSM 15670 induced by 100 µM urolithin C or vehicle were incubated with 100 µM urolithin C or vehicle for 6 h. Data are mean ± SD (n = 3). (*B*, *Left*) Differential gene expression in *Eb* DSM 15670 induced by 100 µM urolithin C or vehicle (n = 3 biological replicates). Genes with adjusted *P* values < 0.05 were colored in navy. (*Right*) Gene annotations of the two gene clusters upregulated in response to urolithin C treatment. (*C*) Phylogenetic tree of biochemically characterized bacterial xanthine oxidase enzymes and Ucdh. Nodes with bootstrap value > 0.7 are shown in green. The outgroup represents eukaryotic xanthine oxidase enzymes. (*D*) Metabolism of urolithin C by *E. coli* TP1000 cell suspension expressing Ucdh alone or Ucdh and XdhC under anaerobic conditions. Data are mean ± SD (n = 3). (*E*) Dehydroxylation of urolithin C by purified Ucdh. Data are mean ± SD (n = 3). The full assay was performed anaerobically by adding 2 µM Ucdh to a pH 7.0 buffer (20 mM MOPS, 300 mM NaCl) containing 100 µM urolithin C and 1 mM NADH. *Eb*, *Enterocloster bolteae.*

We independently set out to identify the enzyme responsible for the C9-OH dehydroxylation. The *E. bolteae* genome encodes 13 proteins annotated as xanthine oxidases (IPR016208) whose amino acid sequence identity to 4-HBCR ranges from 23 to 34%. To determine whether differential gene expression analysis could pinpoint candidate enzymes, we tested whether the urolithin C dehydroxylation activity is induced by substrate. The *E. bolteae* cell suspension from a culture grown with urolithin C showed activity, whereas the suspension from a vehicle-treated culture lacked activity (Fig. 4*A*). Thus, we applied a differential gene expression analysis to *E. bolteae* treated with urolithin C or vehicle and identified two gene clusters (cluster I and cluster II) upregulated in the presence of the substrate (Fig. 4*B*). Cluster I contained functionally diverse protein-encoding genes, including transcriptional regulators, a methyltransferase, and three subunits of a cytoplasmic xanthine oxidase family enzyme (Ucdh): a catalytic subunit harboring the MCD cofactor (UcdhC), a medium subunit with a flavin adenine dinucleotide (FAD) cofactor (UcdhA), and a small subunit with two 2Fe-2S clusters (UcdhB). Notably, 4-HBCR was the closest characterized member of the xanthine oxidase family to Ucdh (32.7% amino acid identity, 49.2% amino acid similarity) (Fig. 4*C*). The gene cluster also encodes a putative transporter that may import urolithins produced and secreted by Coriobacteriia species (Fig. 4*B*). Cluster II encoded proteins involved in molybdate transport and molybdopterin cofactor biosynthesis, maturation, and insertion, including XdhC, a chaperone that aids MCD maturation and insertion for xanthine oxidase family enzymes (61). The prior study also identified Ucdh using similar differential expression analysis (54). Given these results, we hypothesized a role for Ucdh in urolithin C dehydroxylation.

Although Ucdh has been linked to the dehydroxylation of urolithin C to urolithin A, its activity has not been shown in vitro due to its low solubility and incomplete MCD maturation when expressed in a heterologous expression host (54). To confirm its enzymatic activity, we sought to obtain active Ucdh by expressing the protein under anaerobic conditions given the oxygen sensitivity of 4-HBCR (62). Our initial attempt to express Ucdh in *E. coli* BL21 (DE3) yielded inactive protein, presumably due to challenges with MCD maturation and insertion. To overcome this issue, we used *E. coli* TP1000 (63), an engineered host which overproduces the MCD cofactor. TP1000 cell suspensions expressing Ucdh converted urolithin C to urolithin A, and coexpression with the putative chaperone XdhC from cluster II that may assist proper folding of Ucdh further increased urolithin A production (Fig. 4*D*). The Ucdh purified from this expression system (*SI Appendix*, Fig. S3*A*) converted urolithin C to urolithin A with the addition of NADH as an electron donor (Fig. 4*E*). Ucdh also regioselectively dehydroxylated other C9-OH containing urolithins at this position (*SI Appendix*, Fig. S3*B*), including isourolithin A whose C9-OH is not part of a catechol, further illustrating that its activity is distinct from that of catechol dehydroxylases. In summary, we have identified and characterized the xanthine oxidase enzyme Ucdh in vitro as a urolithin C9-OH dehydroxylase.

To understand the distribution of Ucdh homologs in sequenced bacteria, we searched the National Center for Biotechnology Information (NCBI) RefSeq Select protein database using BLAST. High homology hits (>90% amino acid identity) were exclusively identified in the genomes of *E. bolteae* and its relatives like the human gut bacteria *Enterocloster asparagiformis* and *Enterocloster hominis* [formerly known as *Lachnoclostridium pacaense* (64)] (*SI Appendix*, Fig. S3*C* and Table S1) (65). *E. asparagiformis* dehydroxylated urolithin C in culture, suggesting that the Ucdh homologs in these strains are likely active (*SI Appendix*, Fig. S3*E*). All other hits shared less than 60% amino acid identity with Ucdh (*SI Appendix*, Fig. S3*C*). Notably, most species that encode these low homology hits are anaerobes isolated from environments like sludge, marine sediment, and wastewater (*SI Appendix*, Table S1). Whether these environmental Ucdh homologs also dehydroxylate urolithin C is currently unknown. Alternatively, these enzymes may catalyze other reductive transformations related to anaerobic degradation of hydrocarbons and aromatic organic compounds (66–72). Similarly, when we extended our search to a metagenome-assembled genomes dataset that contains >150 k genomes of human-associated microbes, all high homology hits (>90% amino acid identity) were identified in the genomes of *Enterocloster* species (*SI Appendix*, Fig. S3*D*). Altogether, this analysis suggests that the distribution of Ucdh is limited to a few *Enterocloster* species in the human gut.

### Metagenomic Abundance of the *eam* Gene Cluster Correlates with Urolithin A Levels and Gut Inflammation.

**Fig. 5.**
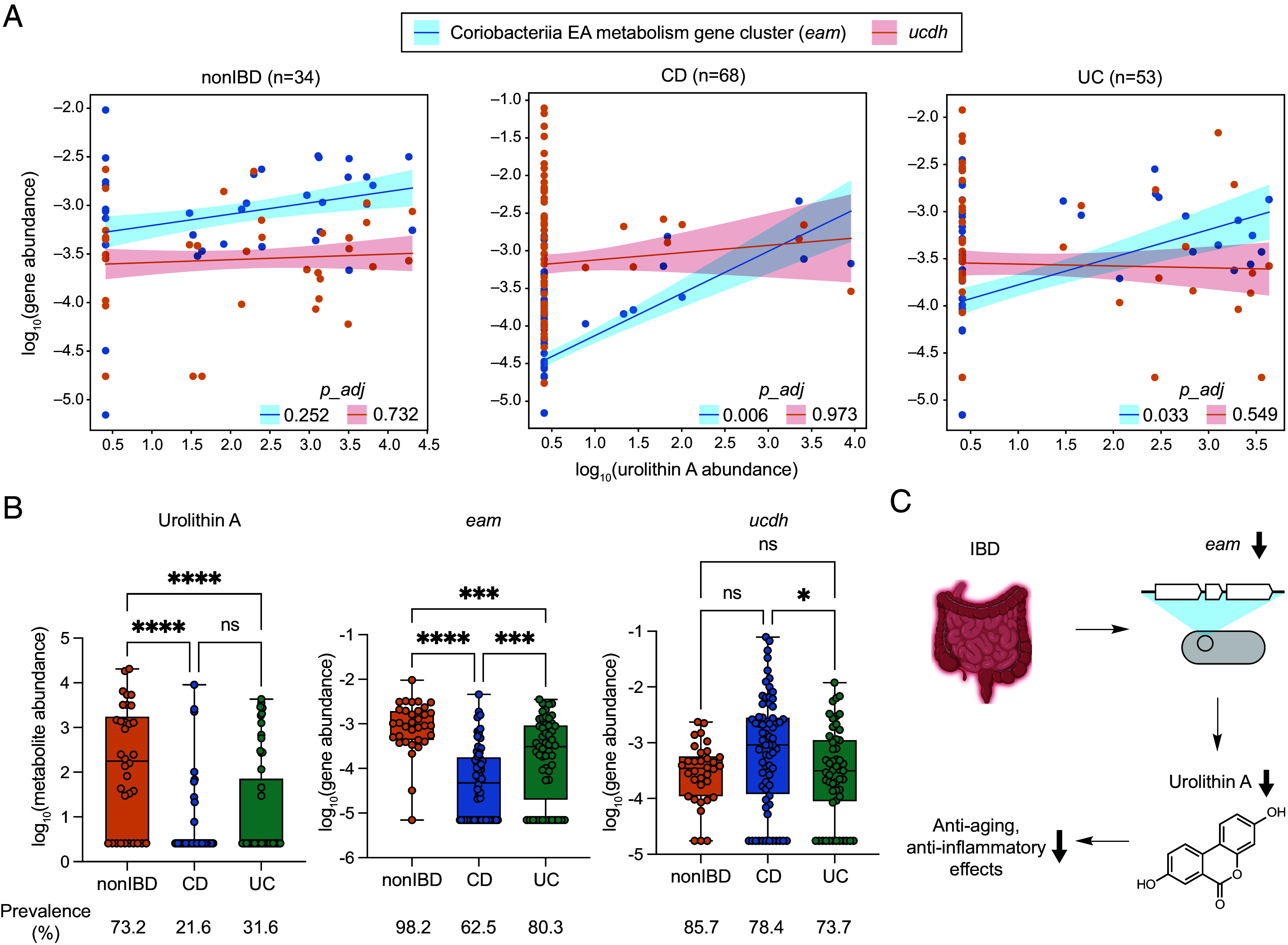
Metagenomic abundance of the *eam* gene cluster correlates with urolithin A levels and gut inflammation. (*A*) Scatter plots showing multivariate linear regression analyses between urolithin A metabolomic abundance and the *eam* gene cluster (average of *eah*, *eadh1*, and *eadh2*) or *ucdh* metagenomic abundances. Lines represent trend, and colored areas represent SE. Adjusted *P* values were calculated using Benjamini–Hochberg correction with a target FDR of 0.05. (*B*) Boxplots of urolithin A concentration, Coriobacteriia gene cluster abundance, and *ucdh* abundance across host phenotypes. One-way ANOVA, Tukey’s multiple comparison. ns, nonsignificant; *0.01 < *P* value < 0.05; ***0.0001 < *P* value < 0.001; ****P value < 0.0001. (*C*) Schematic of a potential mechanistic link connecting IBD, the Coriobacteriia gene cluster, urolithin A, and its health effects. IBD, inflammatory bowel disease; CD, Crohn’s disease; UC, ulcerative colitis.

Having characterized the biochemical activities of urolithin A-producing enzymes in vitro, we sought to evaluate their relevance in vivo by correlating fecal urolithin A levels with urolithin A-producing gene abundance in datasets from clinical studies. We chose to investigate a cross-sectional cohort of Crohn’s disease (CD), ulcerative colitis (UC), and non-inflammatory bowel disease (nonIBD) subjects from the Prospective Registry in IBD Study at MGH (PRISM) (73). This cohort includes paired metagenomic and untargeted metabolomic data for stool samples from 155 subjects. We first quantified the metagenomic abundance of each urolithin A-producing gene (*eah*, *eadh1*, *eadh2*, and *ucdh*) separately. Since *eah*, *eadh1*, and *eadh2* are found in the same gene cluster, we averaged the abundance of these genes to calculate the Coriobacteriia EA metabolism (*eam*) gene cluster abundance. Consistent with the high prevalence of the encoding species in the human gut (73), the *eam* gene cluster and *ucdh* are present in the majority of stool metagenomes from healthy participants (98.2% for the *eam* gene cluster and 85.7% for *ucdh*). Next, we identified the mass peak corresponding to urolithin A in the untargeted metabolomics dataset by coelution of stool metabolite samples with an authentic urolithin A standard (*SI Appendix*, Fig. S4 *A* and *B*). Upon multivariate linear regression analysis with age and medication use as covariates, we found statistically significant positive correlations between the *eam* gene cluster abundance and urolithin A levels for the CD (adjusted *P* = 0.006) and UC subjects (adjusted *P* = 0.033) (Fig. 5*A*). No significant correlation was found in the nonIBD subjects (adjusted *P* = 0.252), potentially due to its small sample size (n = 34). On the other hand, no significant correlations were observed between *ucdh* abundance and urolithin A levels in any cohort (Fig. 5*A*). Though further studies are needed to understand this lack of correlation, our results revealed a possible connection between in vivo urolithin A levels and the Coriobacteriia *eam* gene cluster.

We then compared metabolite and gene abundances between host phenotypes considering inflammation alters the production of gut bacterial metabolites (73–76). Specifically, we postulated that reactive oxygen species generated during gut inflammation may negatively affect EA-metabolizing anaerobic bacteria and enzymes (77, 78). We found that urolithin A levels and the *eam* gene cluster abundance were lower in IBD compared to nonIBD controls (Fig. 5*B*). Additionally, whereas urolithin A was present in 73.2% of nonIBD controls, only 21.6% of CD and 31.6% of UC samples had detectable levels of the metabolite. Furthermore, the *eam* gene cluster was detected more frequently in stool metagenomes from nonIBD subjects (98.2%) compared to CD (62.5%) and UC (80.3%). On the other hand, *ucdh* abundance did not show a significant decrease in IBD subjects. Besides urolithin A, we attempted to correlate EA and several other urolithins, including urolithin B, urolithin C, and isourolithin A, with IBD status. However, these compounds were not found in the metabolomics dataset presumably due to their low abundance or weak mass spectrometry detection. Since urolithin A has anti-inflammatory and anti-aging effects, this reduction in levels of urolithin A and producing enzymes may suggest IBD patients experience reduced health benefits from EA consumption (Fig. 5*C*). Further investigation is required to test the causality of this suggested mechanistic connection.

## Discussion

**Fig. 6.**
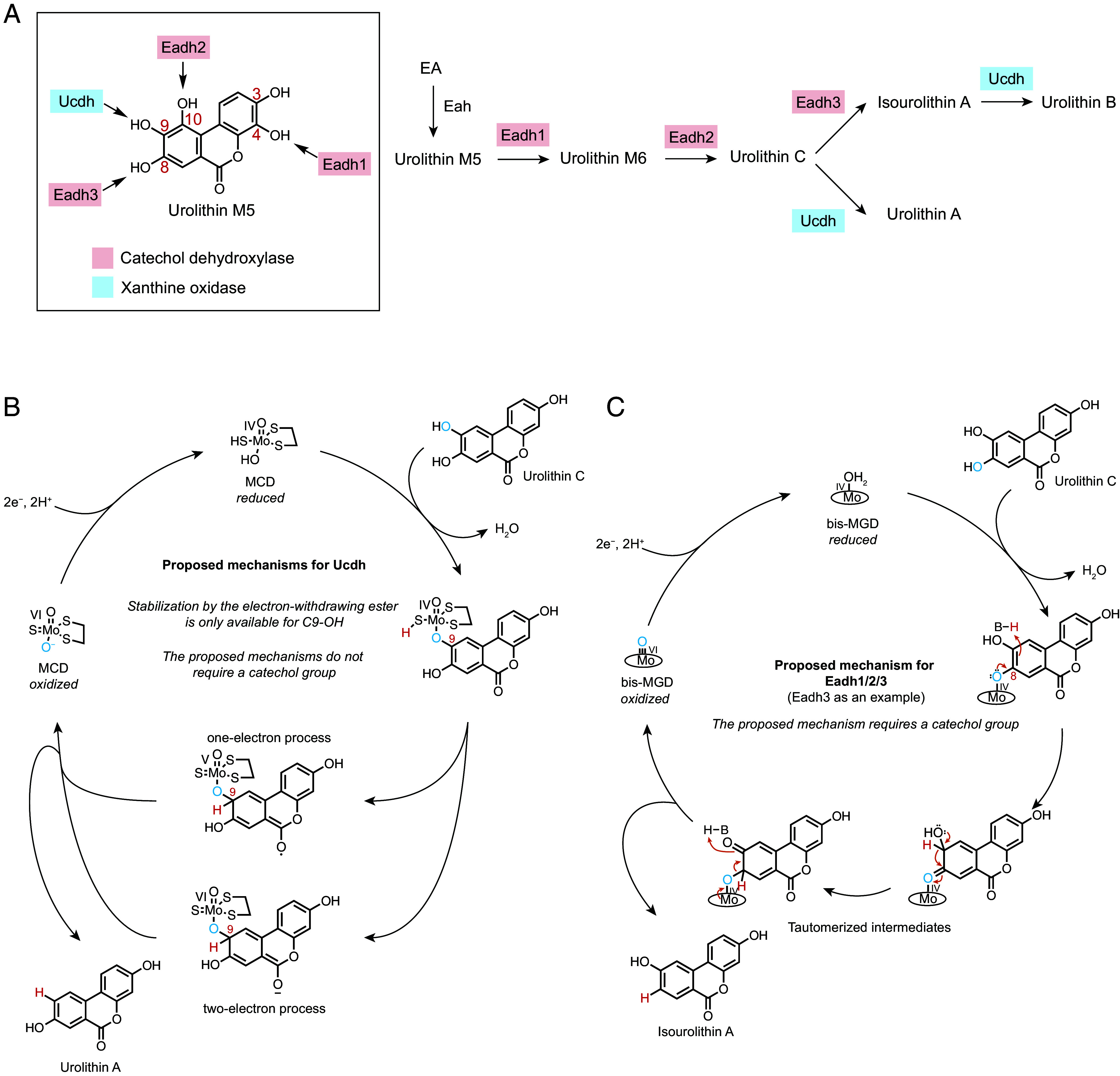
Proposed mechanisms for Ucdh may explain regioselectivity of urolithin dehydroxylation. (*A*) Regioselectivity of Ucdh and Eadh1/2/3 and the assignment of the EA metabolic pathways to urolithin A and urolithin B. (*B*) The lactone functional group of the urolithins may stabilize proposed intermediates during Ucdh catalysis. (*C*) The proposed mechanism of Eadh1/2/3 is consistent with the requirement for a catechol functional group.

In this study, we identified gut bacterial enzymes that produce the anti-inflammatory urolithin A and other urolithins from EA. The conversion of EA to urolithin A represents an example of the two classes of phenol dehydroxylases functioning in tandem (Fig. 6*A*), expanding our fundamental understanding of gut bacterial enzymes involved in phenol dehydroxylation. We showed that several urolithins, including urolithin A and urolithin B, can be produced in vitro from EA through the activity of different combinations of the enzymes that dehydroxylate urolithins at C4, C8, C9, and C10 (Fig. 6*A*). Recently, C3-OH dehydroxylated urolithins, such as the urolithin A isomer urolithin AR, were identified in human stool samples (79). These metabolites are not produced by the dehydroxylases identified in this work or other studies (54, 80), suggesting the presence of as-yet-uncharacterized phenol dehydroxylases targeting the C3-OH of urolithins.

Considering their differing requirements for a catechol motif (Fig. 3*B* and *SI Appendix*, Fig. S3*B*), we suspect that Eadh1/2/3 and Ucdh employ distinct mechanisms to accomplish seemingly similar reactions. The mechanism of Ucdh may be analogous to that of 4-HBCR, for which two potential mechanisms have been proposed: a Birch-type one-electron process (59, 60) and a two-electron process (81) (Fig. 6*B* and *SI Appendix*, Fig. S5*A*). For both mechanisms, the electron-withdrawing thioester group of 4-hydroxybenzoyl-CoA is suggested to play an essential role in stabilizing the anion or radical intermediate generated during catalysis. Notably, C9-OH of the urolithins is the only hydroxyl group on this scaffold positioned *para* to the electron-withdrawing ester functional group, perhaps suggesting substrate electronics play a role in dictating selectivity of dehydroxylation by enzymes from this class (Fig. 6*B*). These proposed mechanisms for Ucdh do not require the departing hydroxyl group to be part of a catechol, consistent with our findings. In contrast, the proposed mechanism for catechol dehydroxylases requires an adjacent hydroxyl group, which undergoes tautomerization to prime the substrate for dehydroxylation (Fig. 6*C* and *SI Appendix*, Fig. S5*B*) (45). Interestingly, a recent report identified a member of the xanthine oxidase enzyme family in *Enterocloster* species other than *E. bolteae* that dehydroxylates C10-OH of urolithins (80). We suspect that its reaction intermediate is stabilized by the *ortho*-phenol through inductive and/or resonance effects. Given that C10-OH is also removed by Eadh2, comparing the mechanisms of these two dehydroxylases presents an exciting direction for future investigation.

Substrate positioning in the active site is likely also a key determinant of regioselectivity in both classes of dehydroxylases. For example, most characterized catechol dehydroxylases remove the sterically less hindered *para* hydroxyl group (Fig. 1*C*). This may be because their catalytic molybdenum cofactor is buried within the protein, requiring substrates to access the cofactor through a narrow funnel (46). Interestingly, Eadh1 and Eadh2 remove sterically hindered hydroxyl groups (C4-OH and C10-OH, respectively), suggesting potential changes in their active sites to accommodate the substrates. Further studies are needed to understand the mechanistic and molecular basis for regioselectivities of the two molybdenum-dependent dehydroxylase classes. Additionally, our kinetic experiments showed a strong substrate preference for each catechol dehydroxylase, explaining the sequence of the metabolic pathway. For example, the >60-fold higher catalytic efficiency of Eadh1 over Eadh2 toward urolithin M5 aligns with the predominant production of urolithin M6 over urolithin D in culture (30, 32). Similarly, previous reports analyzing urolithins in human stool samples did not detect urolithin D (79, 82). Interestingly, urolithin D was observed as a major intermediate in Iberian pigs, suggesting that enzymes encoded in pig microbiomes may have different substrate preferences (58). In summary, our work expands the substrate scope of the catechol dehydroxylase class and connects enzyme kinetic parameters with the observed metabolic pathway of EA in humans.

Our transcriptomic and biochemical experiments linked the critical transformation in urolithin A formation, dehydroxylation of urolithin C C9-OH by *E. bolteae*, to the xanthine oxidase family enzyme Ucdh. We note that another study independently linked this enzyme to urolithin C dehydroxylation using substrate-induced expression (54). However, their functional characterization was limited by the minimal activity and poor solubility of heterologously expressed Ucdh. Here, we addressed this challenge by using the TP1000 *E. coli* strain, which overproduces the MCD cofactor required by xanthine oxidase enzymes. Coexpression of Ucdh with its putative chaperone XdhC significantly improved activity in TP1000 cell suspensions, a strategy that was previously shown to increase activity of a xanthine dehydrogenase (61). After 4-HBCR, Ucdh is the second enzyme found to perform reductive dehydroxylation within the xanthine oxidase family, whose members catalyze mostly oxidative reactions (81). The discovery of Ucdh implies that additional reductive enzymes remain to be uncovered within the xanthine oxidases. For example, a recent study found that human gut species encoding an uncharacterized xanthine oxidase enzyme reduce uric acid to xanthine using the reverse reaction of xanthine dehydrogenase (83). However, this enzyme’s function has not been biochemically characterized. Another recent study identified and characterized a gut bacterial xanthine oxidase homolog that reductively cleaves an aromatic C–S bond in the ergothioneine catabolic pathway (84), further expanding the repertoire of reductive chemistry catalyzed by this enzyme family. Our results, including the anaerobic protein expression system for Ucdh, will enable future efforts to biochemically characterize Ucdh and additional reductive xanthine oxidases.

Previously, our group and the Light group showed that the activities of other catechol dehydroxylases are linked to anaerobic respiration in *Eggerthella* and *Gordonibacter* species (45, 48, 50). Whether the activities of Eadh1/2/3 are linked to anaerobic respiration is currently unknown, and it is possible these enzymes have different biological roles. On the other hand, Ucdh has a different biological role from 4-HBCR which is involved in the catabolism of aromatic compounds. In addition, 4-HBCR accepts reduced ferredoxin as an electron donor, whereas NADH is an electron donor for Ucdh. NAD+ regeneration during fermentation could be a potential biological role of Ucdh. Alternatively, Pidgeon et al. suggested a role for Ucdh in detoxification based on the observation that the catechol-containing substrate of Ucdh (urolithin C) limits iron availability, thereby delaying the growth of *Enterocloster* species (54). Dehydroxylation of urolithin C could rescue the encoding species from growth delay presumably by disrupting iron chelation. Altogether, further studies are needed to elucidate the biological roles of urolithin-dehydroxylating enzymes.

Leveraging our knowledge of the genes and enzymes involved in EA metabolism, we found correlations between in vivo urolithin A levels and the abundance of urolithin A-producing genes in human gut microbiomes. Interestingly, levels of urolithin A were positively correlated with the abundance of the urolithin C-producing Coriobacteriia (*eam*) gene cluster. This correlation may be related to a previously reported positive correlation between *Gordonibacter* species levels and urolithin A production in healthy volunteers, although we only observed the positive correlation in IBD patient samples (28). In contrast, levels of urolithin A were not correlated with *ucdh*, which may arise from discrepancies between in vivo metabolic activity and metagenomic abundance. Alternatively, we cannot rule out the possibility that some individuals might not have consumed EA or the presence of convergently evolved gut bacterial enzymes that produce urolithin A. Finally, we observed a decrease in both urolithin A levels and *eam* gene cluster abundance in the IBD cohort compared to nonIBD controls, suggesting a potential route by which host inflammation alters gut microbiome composition and impacts metabolite production. Lowered urolithin A levels in subjects with IBD may indicate they derived reduced benefits from consuming EA-rich foods. However, we acknowledge that altered levels of urolithin A could also arise from other phenotypic differences, including differences in dietary habits. Mechanistic investigation linking IBD, EA-metabolizing gene levels, and urolithin A production requires further testing in model organisms or human clinical cohorts. These results demonstrate the utility of applying obtained knowledge of gut bacterial metabolic genes and enzymes to human multiomics datasets.

## Materials and Methods

### General Materials and Methods.

All bacterial culturing work was performed in an anaerobic chamber (Coy Laboratory Products) under an atmosphere of 2 to 4% hydrogen, 2 to 4% carbon dioxide, and nitrogen as the balance. The human gut species were grown at 37 °C on Brain–Heart Infusion (BHI) medium or BHI supplemented with 1% L-arginine monohydrochloride (w/v%), 0.05% L-cysteine monohydrochloride (w/v%), and 10 mM sodium formate (BHIrcf medium). The plasmids constructed in this work were deposited on Addgene (Addgene ID 249564–249569).

### Coriobacteriia Screening for EA Metabolizers.

Turbid 48-h starter cultures of Coriobacteriia species in BHI medium were diluted 1:100 into 200 µL of BHIrcf medium supplemented with 100 µM EA in triplicates on a 96-well plate. The plate was then sealed, and the cultures were anaerobically grown at 37 °C for 72 h. The supernatants were analyzed using LC–MS/MS, as described in *SI Appendix*.

### Substrate-Dependent Inducibility Experiment.

(*Gs* 28C) A turbid 48-h starter culture of *Gs* 28C in BHI medium was diluted 1:100 into 5 mL of BHIrcf medium in six replicates. The cultures were anaerobically incubated at 37 °C. When optical density at 600 nm (OD_600_) of the cultures reached 0.2, 50 µL of a 10 mM solution of EA in dimethylformamide (DMF) was added to three replicates (final EA concentration 100 µM), and 50 µL of DMF vehicle was added to the other three replicates. The cultures were further anaerobically incubated at 37 °C until OD_600_ reached 0.5, at which cells were pelleted by centrifugation. In an anaerobic chamber, the cell pellets were washed with 5 mL of 1× phosphate-buffered saline and resuspended in 1 mL of lysis buffer (20 mM Tris, pH 7.5, 500 mM NaCl, 10 mM MgSO_4_, 1 mM CaCl_2_, 0.1 mg/mL DNase, 0.5 mg/mL lysozyme, 1 tablet/50 mL SIGMAFAST protease inhibitor). Cells were lysed via sonication (Branson Sonifier 450, 25% amplitude, 10 s on, 40 s off, 2 min total sonication time). The lysates were then clarified via centrifugation (20 k RCF, 15 min) and were subjected to the activity assay. In a 96-well plate, 1 µL of a 10 mM solution of EA in DMF (100 µM final concentration) or vehicle and 2 µL of 50 mM MV and 50 mM NaDT (1 mM final concentration) were added to 95 µL of cell lysate in triplicates. The plate was then sealed and left in the anaerobic chamber at room temperature for 48 h. The supernatants were analyzed using LC–MS/MS, as described in *SI Appendix*.

(*Eb* DSM 15670) A turbid 48-h starter culture of *E. bolteae* DSM 15670 in BHI medium was diluted 1:100 into 10 mL of BHI medium in six replicates. The cultures were anaerobically incubated at 37 °C. When OD_600_ of the cultures reached 0.3, 100 µL of a 10 mM solution of urolithin C in DMF was added to three replicates (100 µM final concentration), and 50 µL of vehicle was added to the other three replicates. The cultures were further anaerobically incubated at 37 °C until OD_600_ reached 0.9, at which cells were pelleted by centrifugation. In an anaerobic chamber, the cell pellets were washed with 5 mL of 1× phosphate-buffered saline twice and resuspended in 1 mL of 1× phosphate-buffered saline. In a 96-well plate, 1 µL of a 10 mM solution of urolithin C in DMF (100 µM final concentration) or vehicle was added to 99 µL of cell suspension in triplicates. The plate was then sealed and left in the anaerobic chamber at room temperature for 6 h. The supernatants were analyzed using LC–MS/MS, as described in *SI Appendix*.

### RNA Sequencing.

(*Gs* 28C) A turbid 48-h starter culture of *Gs* 28C in BHI medium was diluted 1:100 into 40 mL of BHIrcf medium in six replicates. The cultures were anaerobically incubated at 37 °C. When the OD_600_ of the cultures reached 0.2, 400 µL of a 10 mM solution of EA in DMF (100 µM final concentration) or vehicle was added to the cultures in three replicates. The cultures were further anaerobically incubated at 37 °C until OD_600_ reached 0.5, at which cells were pelleted by centrifugation and resuspended in 500 µL of Trizol.

(*Eb* DSM 15670) A turbid 48-h starter culture of *Eb* DSM 15670 in BHI medium was diluted 1:100 into 10 mL of BHIrcf medium in six replicates. The cultures were anaerobically incubated at 37 °C. When the OD_600_ of the cultures reached 0.5, 100 µL of a 10 mM solution of EA in DMF (100 µM final concentration) or vehicle was added to the cultures in three replicates. The cultures were further anaerobically incubated at 37 °C until OD_600_ reached 0.9, at which cells were pelleted by centrifugation and resuspended in 500 µL of Trizol.

Then, total RNA was extracted and sequenced, and differential gene expression analysis was performed using DESeq2 v1.44.0 (85), as described in *SI Appendix*. Raw RNA-seq data of *Gs* 28C and *E. bolteae* have been deposited in the Sequence Read Archive under BioProject accession number: PRJNA1110272.

### Quantification of Urolithins in *Gs* 28C Supernatant.

A turbid 48-h starter culture of *Gs* 28C in BHI medium was diluted 1:500 into 1 mL of BHIrcf medium supplemented with 100 µM EA to inoculate three replicate cultures. The cultures were anaerobically incubated at 37 °C for 48 h. At multiple time points, 100 µL aliquots were taken, and the OD_600_ of the cultures was measured. Cells were removed from each aliquot by centrifugation at 4,000× g for 10 min. The supernatants were analyzed using LC–MS/MS, as described in *SI Appendix*.

### Protein Expression.

Plasmids for protein expression were constructed, as described in *SI Appendix*. (Ucdh) Chemically competent *E. coli* TP1000 was transformed with 100 ng of pMB-pTrcHis2A-Ucdh-XdhC, and transformants were selected on a LB agar plate supplemented with kanamycin (100 µg/mL) and ampicillin (100 µg/mL). A single colony was picked and inoculated into LB medium supplemented with kanamycin (100 µg/mL) and ampicillin (100 µg/mL) and grown for overnight at 37 °C. The saturated culture was inoculated 1:200 into 2 L of fresh LB medium supplemented with 1 mM sodium molybdate, 2 mM ammonium ferric citrate, and ampicillin (100 µg/mL) in a 2.8 L anaerobic Erlenmeyer baffled flask and grown at 37 °C at 180 rpm for 3 h, at which OD_600_ reached 0.6. The culture was then sparged with nitrogen gas for 30 min, after which 25 mM sodium fumarate and 10 mM sodium nitrate were added. After the additional sparging with nitrogen gas for 30 min, 25 µM IPTG was added, and the flask was tightly capped. The culture was then grown anaerobically at 18 °C for 16 h without shaking. Cell pellets were harvested by centrifugation at 6,000× g for 20 min at 4 °C and used for protein purification and activity assays immediately, as described in *SI Appendix*. (Eah, Eadh1/2/3) Eah was expressed in *E. coli* BL21(DE3), and Eadh1/2/3 were expressed in *G. urolithinfaciens* and used for activity assays, as described in *SI Appendix*.

### Activity Assay of *E. coli* TP1000 Expressing Ucdh in Cell Suspension.

A single colony of TP1000 strains containing either of pTrcHis2A (empty vector), pMB-pTrcHis2A-Ucdh, or pMB-pTrcHis2A-Ucdh-XdhC was picked and inoculated into 5 mL LB medium with 100 µg/mL kanamycin and 100 µg/mL ampicillin and grown for overnight at 37 °C. The saturated cultures were inoculated 1:100 into 10 mL of fresh LB medium supplemented with 1 mM sodium molybdate, 2 mM ammonium ferric citrate, 10 mM sodium nitrate, 25 µM IPTG, and 100 µg/mL ampicillin in 14 mL culture tubes in triplicates. The cultures were brought into an anaerobic chamber and were grown at 37 °C for 24 h under anaerobic conditions. The cell pellets were washed with 1 mL of prereduced 1× phosphate-buffered saline twice and resuspended into 1 mL of the 1× phosphate-buffered saline. In a 96-well plate, 1 µL of a 10 mM solution of urolithin in DMF (100 µM final concentration) or vehicle was added to 99 µL of cell suspension in triplicates. The plate was then sealed and left in the anaerobic chamber at rt for 24 h. The supernatants were analyzed using LC–MS/MS, as described in *SI Appendix*.

### Phylogenetic Analysis.

#### Molybdenum enzymes from Coriobacteriia strains.

Extracted amino acid sequences of proteins annotated as the catalytic subunit of molybdopterin-dependent oxidoreductases from the genomes of Coriobacteriia strains (*Gs* 28C, *G. pamelaeae* 3C, *G. pamelaeae* 7-10-1b, *G. urolithinfaciens* DSM 27213, and *E. isourolithinifaciens* DSM 104140) (total 305 sequences). These sequences were first grouped by 80% amino acid identity into 144 unique sequences using CD-HIT (86). The unique sequences were then aligned using MAFFT-linsi v7.505 (87) and trimmed using trimal (v1.4.1, -gappyout) (88). A maximum-likelihood phylogenetic tree was generated using iqtree2 (v2.1.3, 1,000 ultrafast bootstraps) (89). The tree was visualized using a free web-based tool, iTOL v6.5.8.

#### Xanthine oxidase.

Protein sequences of the catalytic subunit from 61 biochemically characterized xanthine oxidase family enzymes (IPR016208) were downloaded from the UniProt database (accessed 2023/02/14). The protein sequences and Ucdh were then aligned using MAFFT-linsi v7.505 (87) and trimmed using tirmal (v1.4.1, -gappyout) (88). A maximum-likelihood phylogenetic tree was generated using iqtree2 (v2.1.3, 1,000 ultrafast bootstraps) (89). The eukaryotic xanthine oxidases were served as outgroup for a tree of bacterial xanthine oxidases and Ucdh. The tree was visualized using a free web-based tool, iTOL v6.5.8.

#### Metagenome-assembled genome search.

The amino acid sequences of Eah and the catalytic subunits of Eadh1, Eadh2, Ucdh were used as queries for tBLASTn searches (search translated nucleotide databases using a protein query) with an e-value cutoff of <0.0001. The following six MAG datasets sampling microbial sequencing data from diverse environments ranging from mammalian-associated microbes (85, 90–93) to soils and oceans (94) were searched.

#### Catechol dehydroxylase kinetics.

We adapted a continuous absorbance-based enzyme kinetics assay previously developed for catechol dehydroxylases (48), as described in *SI Appendix*.

#### Metagenomic analysis.

The metagenomic dataset from the PRISM study was downloaded from the National Center for Biotechnology Information (PRJNA400072) (73). Host-associated reads, low-quality reads, and adapter sequences were removed using KneadData v0.10.0 (default setting). Using a BLASTX DIAMOND search (e-value cutoff: 0.0001) (95), reads were mapped on the following protein sequences (Eah [*Gs* 28C], Eadh1 [*Gs* 28C], Eadh2 [*Gs* 28C], and Ucdh [*Eb* DSM 15670]). To prevent nonspecific read mapping, we also included UniRef50 sequences of the top 1000 BLAST hits of each query from UniProtKB (assessed Aug 2021) to the query list. Reads with >80% amino acid identity to each sequence were counted. If a read was mapped on multiple proteins, the read was counted as a hit for the protein that had the highest identity, which increases the specificity of the quantification. The number of hits was normalized first by the number of the total reads of each sample and the lengths of the proteins to read per kilobase million (RPKM). For further statistical analyses across samples, RPKM was normalized by average genome size (AGS), which was calculated using MicrobeCensus from each MGX sample (96). Coriobacteriia EA-metabolizing (*eam*) gene cluster abundance was calculated as an average of the gene abundance of *eah*, *eadh1*, and *eadh2*.

#### Validation of urolithin A in metabolomics datasets.

To confirm the presence of urolithin A in stool metabolomics from the PRISM IBD study, a synthetic standard was analyzed alongside reference pool material from the PRISM study using the same LC–MS profiling method (HILIC-neg) employed in the study (73), as described in *SI Appendix*.

#### Multivariate linear regression analysis.

A “statsmodels” python package (v0.14.1) was used to perform multivariate linear regression analysis between the gene abundances and urolithin A levels in paired metagenome and metabolome samples. Zero values were corrected by adding half the smallest nonzero value of each gene or metabolite level. To stabilize variance, abundances were log-transformed. For each IBD phenotype, we modeled the transformed abundance of urolithin A as a function of the *eam* gene cluster and *ucdh* abundances with age as a continuous covariate and four medications (antibiotics, immunosuppressants, mesalamine, and steroids) as binary covariates (ordinary least squares model). Nominal *P* values were adjusted for multiple hypothesis testing with a target FDR of 0.05 using Benjamini–Hochberg correction.

## Supplementary Material

Appendix 01 (PDF)

## Data Availability

RNA-seq data have been deposited in SRA (PRJNA1110272) (97).
