# Supplementary material for: Distinct classes of gut bacterial molybdenum-dependent enzymes produce urolithins
Source: Proc Natl Acad Sci U S A. 2025 Dec 24;122(52):e2501312122. doi: 10.1073/pnas.2501312122 (PMC12771579; doi:10.1073/pnas.2501312122)
Supplement: Supplementary file 1 — Appendix 01 (PDF) [file pnas.2501312122.sapp.pdf]

PNAS

Supporting Information for

Distinct Classes of Gut Bacterial Molybdenum-Dependent Enzymes Produce Urolithins

Minwoo Bae,[1,2] Xueyang Dong,[1] Julian Avila-Pacheco,[3] Quyen D. Nguyen,[1] Fechi Inyama,[1] Vayu Hill-Maini,[1,4] Clary B. Clish,[3] and Emily P. Balskus[1,3,5*]

1. Department of Chemistry and Chemical Biology, Harvard University, Cambridge, MA 02138, USA
2. Present address: Department of Microbial Pathogenesis, Yale University, West Haven, CT 06516
3. Broad Institute of Massachusetts Institute of Technology and Harvard, Cambridge, MA 02142, USA
4. Present address: Department of Bioengineering, Stanford University, Stanford, CA 94305, USA
5. Howard Hughes Medical Institute, Harvard University, Cambridge, MA 02138, USA

*To whom correspondence may be addressed.

Emily P. Balskus
Email: balskus@chemistry.harvard.edu

This PDF file includes:

Supplementary Materials and Methods
Figures S1 to S5
Tables S1 to S5
SI References

Supplementary Materials and Methods

General materials and methods

The following chemicals were used in this study: ellagic acid (EA) (Sigma-Aldrich, catalog# E2250-1G), urolithin M5 (Toronto Research Chemicals Inc., catalog# TRC-U847035-1mg), urolithin M6 (Toronto Research Chemicals Inc., catalog# TRC-U847040-10mg), urolithin D (Toronto Research Chemicals Inc., catalog# TRC-U847020-5mg), urolithin E (Toronto Research Chemicals Inc., catalog# TRC-U847030-10mg), urolithin M7 (Toronto Research Chemicals Inc., catalog# TRC-U847045-2.5mg), urolithin C (Sigma-Aldrich, catalog# SML3047), urolithin A (AstaTech, catalog# Y10574), isourolithin A (AstaTech, catalog# F51950), urolithin B (Sigma-Aldrich, catalog# OTV000001), L-arginine monohydrochloride (Sigma-Aldrich, catalog# A5131-500G), L-cysteine hydrochloride monohydrate (Sigma-Aldrich, catalog# C7880-100G), sodium formate (Sigma-Aldrich, catalog# 247596-100G), methyl viologen (MV) (Sigma-Aldrich, catalog# 856177-1g), sodium dithionite (NaDT) (Sigma-Aldrich, catalog# 157953-5G), sodium molybdate dihydrate (Sigma-Aldrich, catalog# 243655-100G), dimethylformamide (DMF) (Sigma-Aldrich, catalog# 319937-4L), Isopropyl β-D-1-thiogalactopyranoside (IPTG, ultra-pure grade) (Teknova, catalog# I3325), 4-Isopropylbenzoic acid (cumate) (Ambeed, catalog# A255165), kanamycin sulfate (VWR, catalog# 75856-686), ampicillin (IBI scientific, catalog# IB02040), sodium nitrate (Sigma-Aldrich, catalog# S5506), ammonium ferric citrate (Sigma-Aldrich, catalog# F5879), sodium fumarate (Sigma-Aldrich, catalog# F1506), SIGMAFAST protease inhibitor tablets (Sigma-Aldrich, catalog# S8830), DNase (Sigma-Aldrich, catalog# DN25-1G), lysozyme (Sigma-Aldrich, catalog# L6876-5G), β-Nicotinamide adenine dinucleotide reduced form disodium salt trihydrate (NADH) (Research Products International, catalog# N20100-1.0). LC–MS grade acetonitrile and methanol for LC–MS analyses were purchased from Honeywell Burdick and Jackson. LC–MS grade formic acid was purchased from Sigma-Aldrich (catalog# 5330020050). Brain–Heart Infusion (BHI) broth was purchased from Becton Dickinson (catalog# 211060). Luria-Bertani (LB) medium was purchase from Research Products International (catalog# L24060). TRIzol was purchased from Invitrogen (catalog# 15596026).

10-beta competent *E. coli* (New England Biolabs, C3019H) was routinely used for cloning and DNA construction. *Gordonibacter sp.* 28C (*Gs* 28C), *Paraeggerthella hongkongensis* RC2/2 A, and *Eggerthella* strains were obtained from the Peter Turnbaugh lab at the University of California, San Francisco. *Adlercreutzia equolifaciens* DSM 19450, *Senegalimassilia anaerobia* DSM 25959, *Gordonibacter urolithinfaciens* DSM 27213, *Gordonibacter pamelaeae* 7-10-1b, *Ellagibacter isourolithinifaciens* DSM 104140, *Rubneribacter badeniensis* DSM 105129, *Enterocloster bolteae* DSM 15670 (*Eb* DSM 15670), *Enterocloster asparagiformis* DSM 15981, and *Raoultibacter massiliensis* DSM 103407 were purchased from Leibniz Institute DSMZ. *E. coli* TP1000 strain was obtained as a generous gift from Prof. Kurt Warnhoff at Sanford Research.

Bioinformatics analyses were done on the Harvard Faculty of Arts & Sciences Research Computing Cluster.

RNA sequencing

(*Gs* 28C) Total RNA was isolated by first bead beating to lyse cells and then using the Zymo Research Direct-Zol RNA MiniPrep Plus kit (Catalog # R2070) according to the manufacturer's protocol. Illumina cDNA libraries were generated using a modified version of the RNAtag-Seq protocol (1). Briefly, 500 ng of total RNA was fragmented, depleted of genomic DNA, and

dephosphorylated prior to its ligation to DNA adapters carrying 5'-AN8-3' barcodes with a 5' phosphate and a 3' blocking group. Barcoded RNAs were pooled and depleted of rRNA using the RiboZero rRNA depletion kit (Epicentre). These pools of barcoded RNAs were converted to Illumina cDNA libraries in three main steps: (i) reverse transcription of the RNA using a primer designed to the constant region of the barcoded adaptor; (ii) addition of a second adapter on the 3' end of the cDNA during reverse transcription using SmartScribe RT (Clonetech) as described (1); (iii) PCR amplification using primers that target the constant regions of the 3' and 5' ligated adaptors and contain the full sequence of the Illumina sequencing adaptors. cDNA libraries were sequenced on Illumina HiSeq 2500. For the analysis of RNAtag-Seq data, reads from each sample in the pool were identified based on their associated barcode using custom scripts, and up to one mismatch in the barcode was allowed with the caveat that it did not enable assignment to more than one barcode. Barcode sequences were removed from the first read as were terminal Gs from the second read that may have been added by SMARTScribe during template switching.

(*Eb* DSM 15670) Total RNA was extracted using Trizol following manufacturer's instructions (ThermoFisher Scientific, Waltham, MA, USA). RNA samples were quantified using Qubit 2.0 Fluorometer (ThermoFisher Scientific, Waltham, MA, USA) and RNA integrity was checked with 4200 TapeStation (Agilent Technologies, Palo Alto, CA, USA). rRNA depletion sequencing library was prepared by using three probes from QIAGEN FastSelect rRNA 5S/16S/23S Kit (Qiagen, Hilden, Germany), respectively. RNA sequencing library preparation uses NEBNext Ultra II RNA Library Preparation Kit for Illumina by following the manufacturer's recommendations (NEB, Ipswich, MA, USA). Briefly, enriched RNAs are fragmented for 15 minutes at 94 °C. First strand and second strand cDNA are subsequently synthesized. cDNA fragments are end repaired and adenylated at 3'ends, and universal adapters are ligated to cDNA fragments, followed by index addition and library enrichment with limited cycle PCR. Sequencing libraries were validated using the Agilent Tapestation 4200 (Agilent Technologies, Palo Alto, CA, USA), and quantified using Qubit 2.0 Fluorometer (ThermoFisher Scientific, Waltham, MA, USA) as well as by quantitative PCR (KAPA Biosystems, Wilmington, MA, USA). The sequencing libraries were multiplexed and clustered onto 1 lane of a flowcell. After clustering, the flowcell was loaded onto the Illumina HiSeq instrument (4000 or equivalent) according to manufacturer's instructions. The samples were sequenced using a 2x150bp Paired End (PE) configuration. Image analysis and base calling were conducted by the Illumina Control Software. Raw sequence data (.bcl files) generated was converted into fastq files and de-multiplexed using Illumina bcl2fastq 2.17 software. One mis-match was allowed for index sequence identification.
InterProScan (2) and Gaia (3) were to annotate the up-regulated genes. Raw RNA-seq data of *Gs* 28C and *E. bolteae* have been deposited in the Sequence Read Archive under BioProject accession number: PRJNA1110272.

Differential expression analysis
Kneaddata v0.10.0 (--bypass-trf) was used to clean up raw sequencing data by removing short or poor-quality reads and clipping off Illumina sequencing adaptor sequences. Bowtie2 v2.5.1 (4) and HTSeq 2.0 v0.11.2 (5) were used to align reads to the genome of the sequenced organisms and to count the number of mapped reads onto each protein-encoding gene. DESeq2 v1.44.0 (6) was used to compute fold change, mean of normalized counts, and adjusted p-value of each protein-encoding gene. Variance among genes with low expression levels was stabilized by applying lfcShrink (coef=2, type="apeglm") (7). The plotMA function was used to visualize the results (8).

Plasmid construction

Plasmid construction was carried out using standard molecular biology techniques. (Eah) To construct a plasmid for Eah expression, we first amplified the Eah gene from *Gs* 28C gDNA using primers oMB-Eah-F and oMB-Eah-R (with Gibson Assembly overhangs) and the vector backbone from a pET28a vector using primers oMB-pET28a-F and pMB-oET28a-R. The vector backbone was then gel purified and DpnI digested. The amplified gene fragment and the vector backbone were ligated using Gibson Assembly (pMB-pET28a-Eah). The primers were designed to introduce an N-terminal His_6 tag into Eah. (Eadh1, Eadh2, Eadh3) To construct a cumate-inducible expression vector for catechol dehydroxylases in *G. urolithinfaciens*, we first linearized a previously prepared vector pXD80GHcdh3-pXD68Kan2-Pct3 using a pair of restriction enzymes NsiI and SpeI. The *G. urolithinfaciens* genomic regions containing protein-encoding genes for Eadh1 and Eadh2 subunits, with an insertion of a sequence encoding N-terminal His_6 tag to subunit A, were synthesized by Genewiz. Likewise, the *E. isourolithinifaciens* genomic region containing Eadh3 subunits, with an insertion of a sequence encoding N-terminal His_6 tag into subunit A, was synthesized by Genewiz. Gibson assembly overhang sequences were introduced by amplifying the synthesized gene sequences using primer pairs oMB-Eadh1-F/R, oMB-Eadh2-F/R, oMB-Eadh3-F/R for Eadh1, Eadh2, and Eadh3, respectively. The amplified gene fragments were inserted into the linearized pXD68Kan2 backbone using Gibson Assembly (pMB-pXD80-Eadh1, pMB-pXD80-Eadh2, pMB-pXD80-Eadh3). (Ucdh) To construct a plasmid for Ucdh expression, we first linearized pTrcHis2A using a pair of restriction enzymes BamHI and HindIII. Ucdh subunits were amplified from *E. bolteae* gDNA as two gene fragments using primer pairs oMB-Ucdh-F/oMB-Ucdh-His-R and oMB-Ucdh-His-F/oMB-Ucdh-noXdhC-R. The primers were designed to insert a sequence encoding N-terminal His_6 tag into subunit C The two amplified gene fragments and the linearized pTrcHis2A backbone were ligated using Gibson Assembly (pMB-pTrcHis2A-Ucdh). To construct a plasmid for Ucdh and XdhC co-expression, Ucdh subunits were amplified from pMB-pTrcHis2A-Ucdh using a primer pair oMB-Ucdh-F/R, and XdhC was amplified from *E. bolteae* gDNA using a primer pair oMB-XdhC-F/R. The amplified gene fragments and the linearized pTrcHis2A backbone were ligated using Gibson Assembly (pMB-pTrcHis2A-Ucdh-XdhC).

To prepare electrocompetent *G. urolithinfaciens* cells for plasmid transformation, a turbid 48-hour starter culture of *G. urolithinfaciens* strain in BHI medium was inoculated 1:50 into 40 mL of BHI medium supplemented with 1% L-arginine monohydrochloride (w/v%). When the culture reached optical density at 600 nm (OD_{600}) of 0.3, the cells were pelleted by centrifugation, and the supernatant was removed. The cell pellet was washed with 10 mL of ice-cold sterile water three times and then with 5 mL of deoxygenated sterile 10% (v/v) glycerol aqueous solution. Finally, the cell pellet was resuspended in 2.5 mL of deoxygenated sterile 10% (v/v) glycerol solution and subdivided into 100 µL aliquots, which were flash frozen and stored at –80 °C until use.

To prepare electrocompetent *E. coli* TP1000 cells for plasmid transformation, a turbid overnight culture of *E. coli* strains in LB medium was inoculated 1:100 into 100 mL of LB medium. When the culture reached OD_{600} of 0.4–0.6, the cells were pelleted by centrifugation at 4 °C, and the supernatant was removed. The cell pellet was washed with 40 mL of ice-cold sterile 10% (v/v) glycerol aqueous solution five times. Finally, the cell pellet was resuspended in 1 mL of sterile 10% (v/v) glycerol solution and subdivided into 50 µL aliquots, which were flash frozen and stored at –80 °C until use.

Expression of Eah in E. coli BL21(DE3).

The chemically competent *E. coli* BL21(DE3) was transformed with 100 ng of pMB-pET28a-Eah, and transformants were selected on a LB agar plate supplemented with kanamycin (50 µg/mL). A

single colony was picked and inoculated into LB medium supplemented with kanamycin (50 µg/mL) and grown for overnight at 37 °C. The saturated culture was inoculated 1:200 into 1 L of fresh LB medium supplemented with kanamycin (50 µg/mL) in a 2.8 L Erlenmeyer baffled flask and grown at 37 °C at 180 rpm for 3 hours, at which OD_{600} reached 0.5. IPTG was added to the final concentration of 0.25 mM. The culture was then grown at 18 °C at 180 rpm for 16 hours. Cell pellets were harvested by centrifugation at 6,000 × *g* for 20 min at 4 °C and used for protein purification immediately.

Expression of catechol dehydroxylases in G. urolithinfaciens.
For transformation, electrocompetent *G. urolithinfaciens* cells were electroporated using a MicroPulser Electroporator (BioRad) with 100–1,000 ng of pMB-pXD80-Eadh1/2/3 at 2.5 kV voltage in 1-mm gap width electroporation cuvettes (VWR). 1 mL of pre-reduced BHI medium supplemented with 1% L-arginine monohydrochloride (w/v%) and 10 mM sodium formate was immediately added to the electroporated cells and transferred to 1.7 mL Eppendorf tubes. These tubes were brought to anaerobic chamber (Coy Laboratory Products) and incubated at 37 °C for 3 hours. The cultures were then plated onto BHI agar plates supplemented with 1% L-arginine monohydrochloride (w/v%), 10 mM sodium formate, and kanamycin (100 µg/mL) and grown anaerobically for 3–5 days at 37 °C. A colony was picked and inoculated into BHIrcf medium with kanamycin (100 µg/mL) and grown for 2 days at 37 °C anaerobically. The saturated culture was inoculated 1:25 into 2 L of fresh BHIrcf medium with kanamycin (100 µg/mL) and cumate (100 µM) in a 2.8 L anaerobic Erlenmeyer baffled flask. The culture was grown at 37 °C anaerobically for 20–24 hours without shaking. Cell pellets were harvested by centrifugation at 6,000 × g for 20 min at 4 °C and used for protein purification immediately.

Expression of Ucdh in E. coli TP1000
Electrocompetent *E. coli* TP1000 was transformed with 100 ng of pMB-pTrcHis2A-Ucdh-XdhC, and transformants were selected on a LB agar plate supplemented with kanamycin (100 µg/mL) and ampicillin (100 µg/mL). A single colony was picked and inoculated into LB medium supplemented with kanamycin (100 µg/mL) and ampicillin (100 µg/mL) and grown for overnight at 37 °C. The saturated culture was inoculated 1:200 into 2 L of fresh LB medium supplemented with 1 mM sodium molybdate, 2 mM ammonium ferric citrate, and ampicillin (100 µg/mL) in a 2.8 L anaerobic Erlenmeyer baffled flask and grown at 37 °C at 180 rpm for 3 hours, at which OD_{600} reached 0.6. The culture was then sparged with nitrogen gas for 30 min, after which 25 mM sodium fumarate and 10 mM sodium nitrate were added. After the additional sparging with nitrogen gas for 30 min, 25 µM IPTG was added, and the flask was tightly capped. The culture was then grown anaerobically at 18 °C for 16 hours without shaking. Cell pellets were harvested by centrifugation at 6,000 × *g* for 20 min at 4 °C and used for protein purification immediately.

Enzyme Purification

All subsequent steps were performed at 4 °C. Eah was purified aerobically whereas catechol dehydroxylases and Ucdh were purified anaerobically. Cell pellets were resuspended at 5 mL per g cell paste in lysis buffer (50 mM HEPES, 30 mM imidazole, 250 mM NaCl, 2 mg/mL lysozyme, pH 8). Cells were lysed by sonication using a ½ inch horn at 35% amplitude for 6 min (10 sec on followed by 40 sec off) while being kept in an ice water bath. Lysate was centrifuged at 14,000 × *g* for 50 min to separate soluble and insoluble fractions. 2 mL bed volume of Ni-NTA resin was equilibrated with lysis buffer and was then mixed with soluble lysate followed by 1 hour incubation with constant agitation. The lysate was then loaded onto a column by gravity flow. The column was washed with 10 column volumes of wash buffer (50 mM HEPES, 30 mM imidazole, 250 mM

NaCl, pH 8). Protein was eluted from the column with elution buffer (50 mM HEPES, 250 mM imidazole, 250 mM NaCl, pH 8). Combined elution fractions containing protein were concentrated using an Ultra-15 Centrifugal Filters centrifugal concentrator with a 30 kDa MWCO membrane (Amicon) and desalted using a disposable PD-10 desalting column (cytiva). The desalted proteins were again concentrated using a new centrifugal concentrator with a 30 kDa MWCO membrane (Amicon). Protein concentrations were estimated using a NanoDrop 2000 UV-Vis Spectrophotometer (Thermo Scientific) using extinction coefficient at a wavelength of 280 nm (ε280) calculated by ExPASY ProtParam as follows: Eadh1A(NHis$_6$)B (ε280 = 227,965 M^{-1} cm^{-1}), Eadh2A(NHis$_6$)B (ε280 = 215,725 M^{-1} cm^{-1}), Eadh3A(NHis$_6$)B (ε280 = 229,955 M^{-1} cm^{-1}). Protein concentrations of Eah(NHis$_6$) and UcdhABC(NHis$_6$) were calculated using a Bradford assay.

Enzyme activity assays

All assays were performed in an anaerobic chamber containing N_2 and <0.1 ppm O_2 (MBRAUN). All assay concentrations are final concentrations.

Eah activity assay
1 µM Eah or blank enzyme storage buffer was added in triplicate to assay buffer containing 100 µM EA (50 mM HEPES, 250 mM NaCl, pH 7.0). The final reaction solution was sealed and left at rt, without shaking, for 20 hours. For LC–MS/MS analysis, 40 µL of the solution was first diluted 1:5 into LC–MS grade methanol, followed by centrifugation to remove precipitates before injection onto LC–MS/MS, as described below.

Catechol dehydroxylase activity assay
100 nM of each catechol dehydroxylase or blank enzyme storage buffer was added in triplicate to assay buffer containing 200 µM MV, 100 µM NaDT, and 100 µM substrate (20 mM MOPS, 300 mM NaCl, pH 7.0). The final reaction solution was sealed and left at rt, without shaking, for 20–24 hours. For LC–MS/MS analysis, 40 µL of the solution was first diluted 1:5 into LC–MS grade methanol, followed by centrifugation to remove precipitates before injection onto LC–MS/MS, as described below.

Ucdh activity assay
2 µM Ucdh or blank enzyme storage buffer was added in triplicate to assay buffer containing 1 mM NADH and 100 µM substrate (20 mM MOPS, 300 mM NaCl, pH 7.0). The final reaction solution was sealed and left at rt, without shaking, for 20–24 hours. For LC–MS/MS analysis, 40 µL of the solution was first diluted 1:5 into LC–MS grade methanol, followed by centrifugation to remove precipitates before injection onto LC–MS/MS, as described below.

LC–MS/MS methods

Method A (all metabolites except urolithin B)
Samples were analyzed using a Waters UPLC-QQQ Mass Spectrometer equipped with a CORTECS T3 column (Waters Corp.). The following chromatography conditions were used: Column temperature, 40 ºC; Mobile phase A, water (0.1% formic acid); Mobile phase B, acetonitrile (0.1% formic acid); Gradient (percentage denotes the ratio of mobile phase A): 100%, 1 min; 100% to 40%, 1.5 min; 40% to 10%, 0.2 min; 10%, 0.3 min, 10% to 100%, 0.05 min; 100%, 0.35 min; Flowrate, 0.5 mL/min; Injection volume: 1.0 µL.

Method B (urolithin B)

Samples were analyzed using a Waters UPLC-QQQ Mass Spectrometer equipped with a ACQUITY UPLC BEH amide column (Waters Corp.). The following chromatography conditions were used: Column temperature, 40 ºC; Mobile phase A, water (0.1% formic acid); Mobile phase B, acetonitrile (0.1% formic acid); Gradient (percentage denotes the ratio of mobile phase A): 10%, 0.5 min; 10% to 100%, 1.5 min; 100%, 0.5 min; 100% to 10%, 0.25 min; 10%, 0.65 min; Flowrate, 0.5 mL/min; Injection volume: 1.0 µL.

For MS/MS, the precursor and daughter ions were detected via Electrospray Ionization (ESI) in negative mode. Isomers with same MS/MS fragments were distinguished by comparing their retention times to the synthetic standards. EA and urolithins were quantified using synthetic standards when available. The transitions and collision energy used to detect specific compounds of interest are listed. (1) EA: precursor ion *m/z*, 300.85; daughter ion *m/z*, 228.85; collision energy, 28 V; cone voltage, 92 V. (2) Urolithin M5: precursor ion *m/z*, 275.00; daughter ion *m/z*, 257.17; collision energy, 24 V; cone voltage, 82 V. (3) Urolithin M6, urolithin E and urolithin D: precursor ion *m/z*, 258.94; daughter ion *m/z*, 212.95; collision energy, 26 V; cone voltage, 92 V. (4) Urolithin C and urolithin M7: precursor ion *m/z*, 243.01; daughter ion *m/z*, 187.00; collision energy, 28 V; cone voltage, 30 V. (5) Urolithin A and isourolithin A: precursor ion *m/z*, 227.01; daughter ion *m/z*, 198.06; collision energy, 30 V; cone voltage, 52 V, (6) Urolithin B: precursor ion *m/z*, 211.21; daughter ion *m/z*, 167.09; collision energy, 24 V; cone voltage, 42 V

Catechol dehydroxylase kinetics

All assay concentrations are final concentrations. Anoxic catechol dehydroxylases (Eadh1, Eadh2, or Eadh3) and solid substrates were brought into an anaerobic chamber containing N_2 and <0.1 ppm O_2 (MBRAUN). Substrates were dissolved in pre-reduced dimethylformamide (DMF). Each reaction contained 200 µM MV, 100 µM NaDT, 2-fold serial dilutions of urolithins (3.9 to 500 µM), and an enzyme in the anoxic buffer (50 mM MOPS, pH 7.0, 300 mM NaCl). Enzyme concentrations were varied based on enzyme–substrate pairs: 10 nM for Eadh1–urolithin M5, 20 nM for Eadh1–urolithin E, and 200 nM for the other pairs. The initial rate over the first minute was measured using a continuous spectrophotometric assay. In this assay, the reductive dehydroxylation was coupled with the oxidation of $MV^{+\cdot}$ (blue, absorption maxima at 605 nm wavelength) to MV^{2+} (colorless). Each assay was performed using two independently prepared protein samples, with each sample tested in biological triplicates. Kinetic parameters were calculated using GraphPad Prism 10 (GraphPad Software) by fitting to the model $v = (k_{cat} * [E]_0 * [S]) / (K_m + [S])$.

LC–MS methods for validating urolithin A in metabolomics datasets

Metabolites were extracted from PRISM stool homogenate (30 µL) with four volumes of 80% methanol containing inosine-$^{15}N_4$, thymine-d_4 and glycocholate-d_4 internal standards (Cambridge Isotope Laboratories). The extract was centrifuged (10 min, 9,000 *g*, 4 °C), and the supernatant was injected alongside 2 ng of urolithin A onto a 150 × 2.0-mm Luna NH_2 column (Phenomenex). The column was eluted at a flow rate of 400 µL/min with initial conditions of 10% mobile phase A (20 mM ammonium acetate and 20 mM ammonium hydroxide in water) and 90% mobile phase B (10 mM ammonium hydroxide in 75:25 v/v acetonitrile/methanol) followed by a 10-min linear gradient to 100% mobile phase A. MS analyses were carried out using electrospray ionization in the negative ion mode using full scan analysis over *m/z* 60–750 at 70,000 resolution and 3 Hz data acquisition rate. Additional MS settings are: ion spray voltage, −3.0 kV; capillary temperature, 350 °C; probe heater temperature, 325 °C; sheath gas, 55; auxiliary gas, 10; and S-lens RF level 40. Product ion spectra MS/MS was generated with the same parameters at five

different collision energies (HCD: 10, 20, 30, 40, 50 eV), and the *m/z* and retention time of the urolithin A reference standard were used to confirm the identity of the unknown feature in the PRISM data (Study Compound ID: HILn_PRISM_m12047, *m/z*: 227.0348, retention time: 2.78 min).

Supplemental Figures

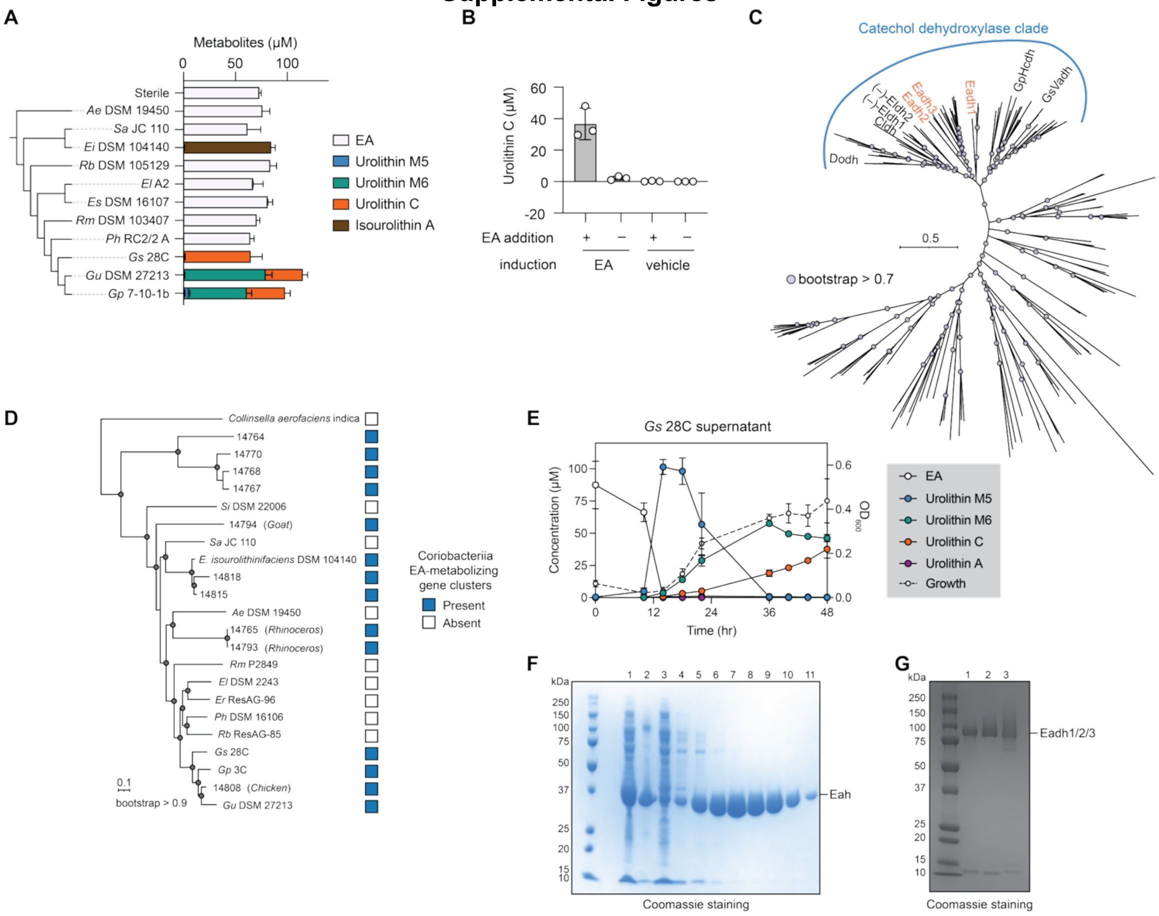

Figure S1. Identification of a Coriobacteriia gene cluster responsible for EA metabolism (A) Endpoint concentrations of EA and urolithins in liquid cultures of Coriobacteriia strains anaerobically grown in medium containing 100 µM EA at 37 °C for 72 h. **(B)** Cell lysates of *Gs* 28C induced by 100 µM EA or vehicle were incubated with either 100 µM EA or vehicle at rt for 24 h. **(A–B)** Data represented as mean ± SD with n = 3 biological replicates. **(C)** Maximum-likelihood phylogenetic tree of molybdenum-dependent oxidoreductases encoded by *Gp* 3C, *Gp* 7-10-1b, *Gu* DSM 27213, *Gs* 28C, and *Ei* DSM 104140. Previously reported catechol dehydroxylases are labelled. Grey nodes indicate bootstrap value > 0.7. **(D)** Phylogenetic distribution of the *eam* gene cluster among animal gut-associated Coriobacteriia. Grey nodes indicate bootstrap value > 0.9. **(E)** Urolithins measured in the supernatants of *Gs* 28C liquid cultures incubated with 100 µM EA at 37 °C for 48 h under anaerobic conditions. Data represented as mean ± SD with n = 3 biological replicates. **(F)** SDS-PAGE image of fractions from Eah protein expression and purification. Lane annotations: 1, lysate; 2, pellet; 3, flow-through; 4–6, wash; 7–11, elution. **(G)** SDS-PAGE image of elution fractions of Eadh1 (lane 1), Eadh2 (lane 2), and Eadh3 (lane 3).

Ae, Adlercreutzia equolifaciens; *Sa, Senegalimassilia anaerobia*; *Ei, Ellagibacter isourolithinifaciens*; *Rb, Rubneribacter badeniensis*; *El, Eggerthella lenta*; *Es, Eggerthella sinensis*; *Rm, Raoultibacter massiliensis*; *Ph, Paraeggerthella hongkongensis*; *Gs, Gordonibacter species*; *Gu, Gordonibacter urolithinfaciens*; *Gp, Gordonibacter pamelaeae*; *Si, Slackia isoflavoniconvertens*; *Er, Enteroscipio rubneri*; Dodh, DOPAC dehydroxylase; Cldh, catechol lignan dehydroxylase; (–)-Eldh1, (–)-dihydroxyenterolactone dehydroxylase 1; (–)-Eldh2, (–)-dihydroxyenterolactone dehydroxylase 2; *Gp* Hcdh, *Gordonibacter pamelaeae* hydrocaffeic acid dehydroxylase; *Gs* Vadh, *Gs* 28C 5-(3',4'-dihydroxyphenyl)valeric acid dehydroxylase.

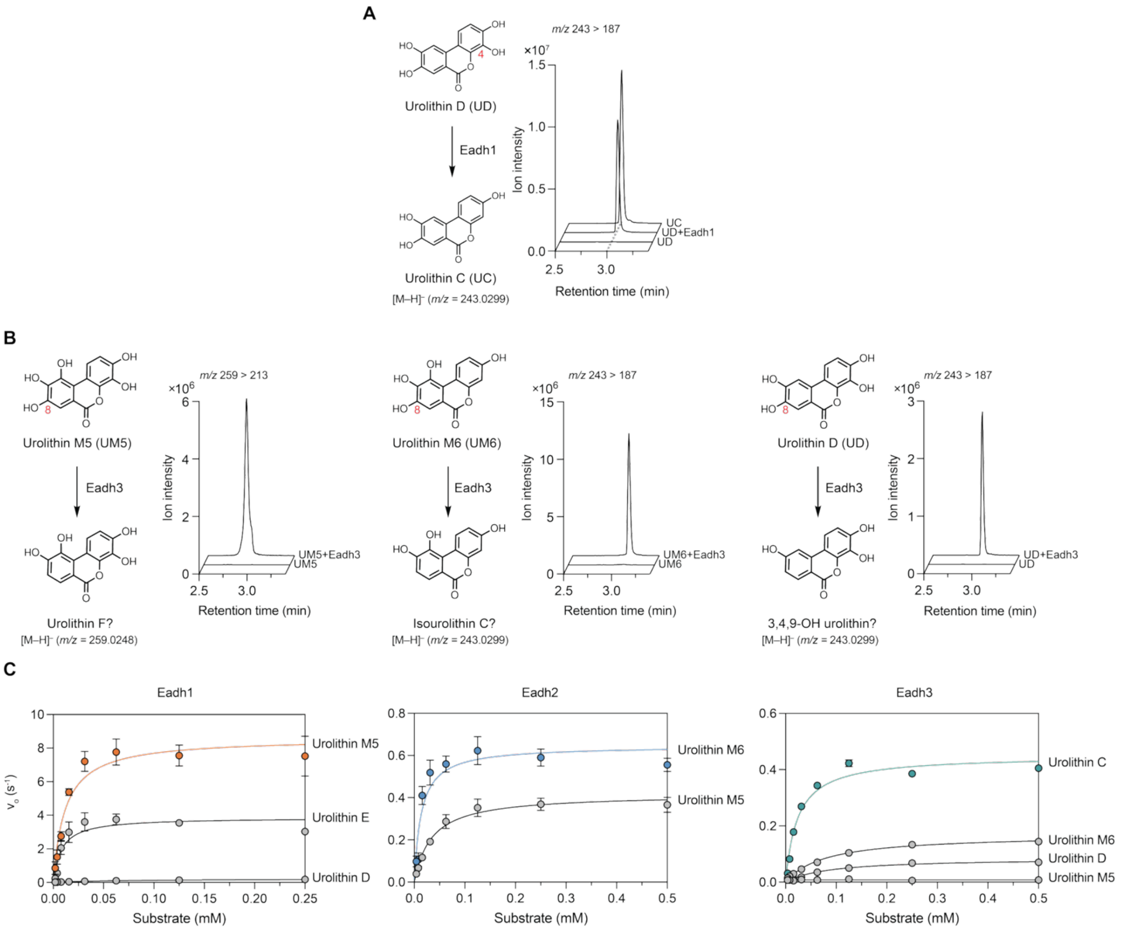

Figure S2. Catechol dehydroxylase substrate specificity and kinetics explain urolithin production. (A) LC–MS/MS traces of urolithin D dehydroxylation by Eadh1. **(B)** LC–MS/MS traces of urolithin metabolism by Eadh3 toward urolithins containing C8-OH. Structures of predicted products are shown. The identities of the products have not been verified due to the lack of available standard compounds. **(A–B)** 100 nM enzyme was incubated with 100 μM substrate, 200 μM methyl viologen, and 100 μM sodium dithionite at rt for 20 h in a pH 7.0 buffer (20 mM MOPS and 300 mM NaCl) under anaerobic conditions. **(C)** Michaelis–Menten curves of Eadh1, Eadh2, and Eadh3 towards various substrates. The reactions were performed anaerobically in a pH 7.0 buffer (20 mM MOPS and 300 mM NaCl) containing varying concentrations of enzymes and substrates, 200 μM methyl viologen, and 100 μM sodium dithionite at 30 °C. Data represented as mean ± SD with n = 6 biological replicates.

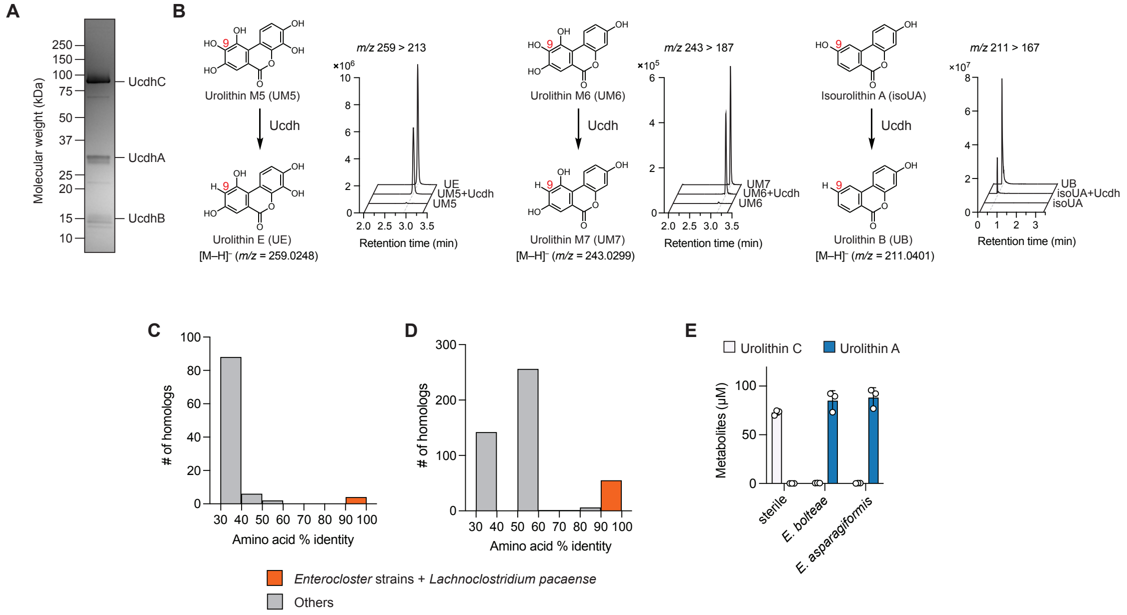

Figure S3. A xanthine oxidase homolog catalyzes dehydroxylation of urolithin C to urolithin A. (A) SDS-PAGE image of an elution fraction of Ucdh. **(B)** LC–MS/MS traces of dehydroxylated products after C9-OH containing urolithins were incubated with Ucdh. 2 μM Ucdh was incubated with 100 μM substrate and 1 mM NADH at rt for 20 h in a pH 7.0 buffer (20 mM MOPS and 300 mM NaCl) under anaerobic conditions. **(C)** A histogram listing top 100 hits with highest amino acid percent identity to Ucdh from the NCBI RefSeq Select protein database. **(D)** A histogram listing Ucdh homologs found in a human-associated microbial assembled genome dataset. **(E)** Urolithin C dehydroxylation to urolithin A by Ucdh-encoding species. Cultures were grown with 100 μM urolithin C at 37 °C for 72 h. Data represented as mean ± SD with n = 3 biological replicates.

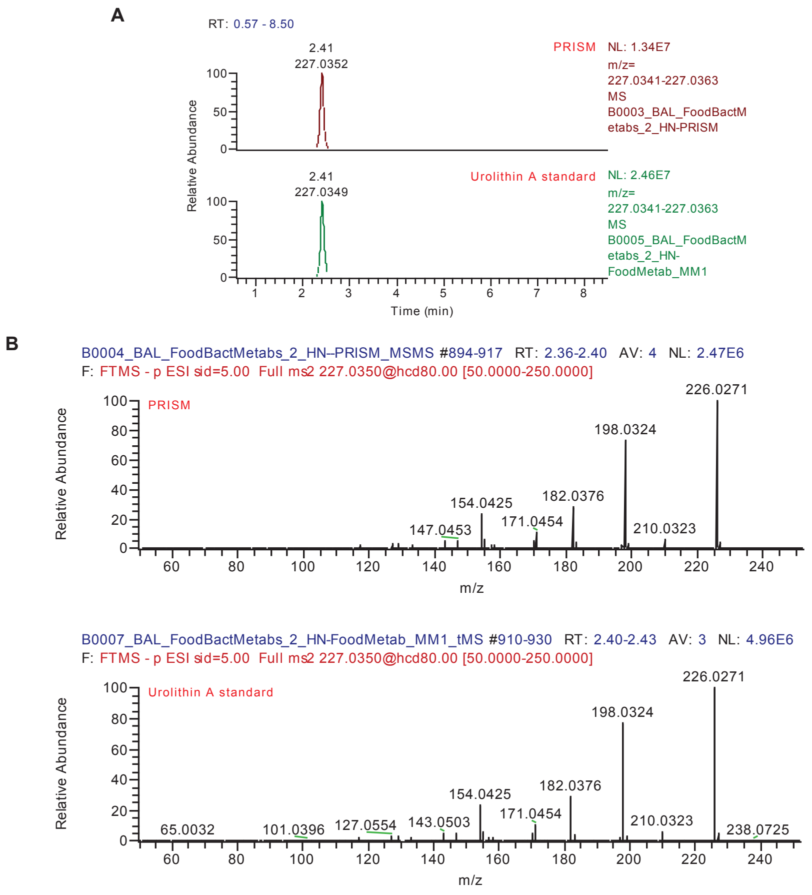

Figure S4. Identification of urolithin A in PRISM metabolome database. (A) Extracted ion chromatogram (EIC) of urolithin A standard (bottom) and a corresponding mass peak (QI1103) in PRISM metabolome database (top). **(B)** MS/MS spectra of urolithin A standard (bottom) and QI1103 (top).

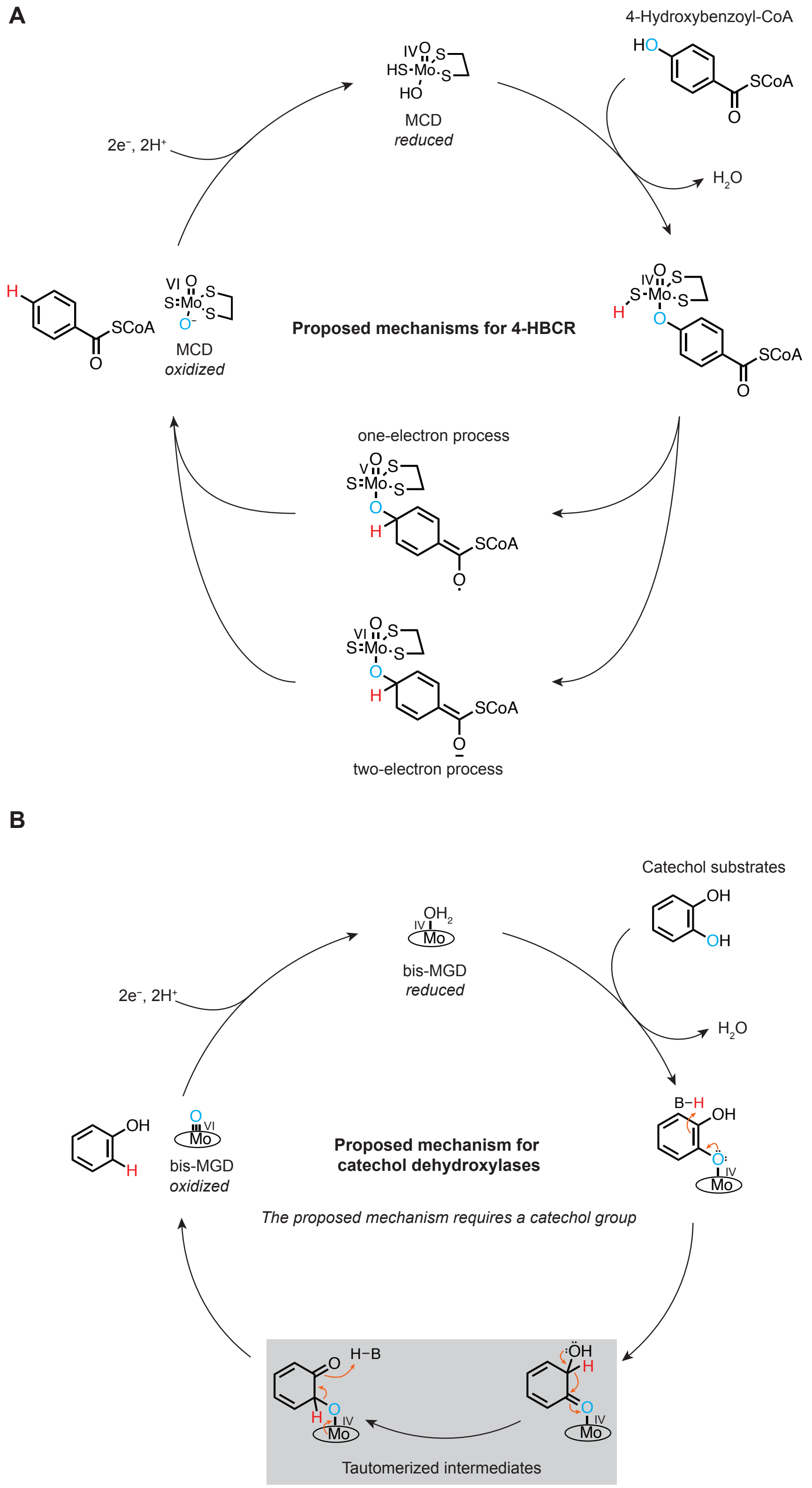

Figure S5. Proposed mechanisms for 4-HBCR and catechol dehydroxylases. (A) Two proposed mechanisms for 4-HBCR. **(B)** Proposed mechanism for catechol dehydroxylases via tautomerization.

Table S1. Top 30 highest homology hits of Ucdh among the NCBI RefSeq Select protein database and their encoding species. UNK, unknown.

Protein ID	%ID	%query coverage	Species	Environment	Anaerobe	Anaerobic catabolism
WP_002575457.1	99.87	100	*Enterocloster bolteae*	Human gut	Y	
WP_025486543.1	97.33	100	*Lachnoclostridium pacaense*	Human gut	Y	
WP_007860285.1	96.44	100	*Enterocloster citroniae*	Human gut	Y	
WP_007711966.1	96.32	100	*Enterocloster asparagiformis*	Human gut	Y	
WP_008711368.1	54.09	97	*Cloacibacillus evryensis*	Anaerobic sludge digester	Y	
WP_207734944.1	53.62	92	*Zhenpiania hominis*	Human gut	UNK	
WP_028319952.1	42.38	95	*Desulfatiglans anilini*	Marine sediment	Y	Aniline, catechol, phenol, benzoate
WP_207682456.1	41.71	96	*Desulfonema magnum*	Marine sediment	Y	*p*-cresol, 3-phenylpropionate
WP_014809669.1	41.46	95	*Desulfomonile tiedjei*	Sewage sludge	Y	
WP_155322555.1	40.77	95	*Desulfosarcina ovata*	Tidal sediment	Y	Toluene
WP_155307404.1	40.66	95	*Desulfosarcina widdelii*	Marine sediment	Y	Hydrocarbon
WP_155319571.1	40	96	*Desulfosarcina alkanivorans*	Marine sediment	Y	Hydrocarbon, *p*-xylene
WP_236006024.1	39.9	96	*Paradesulfitobacterium ferrireducens*	Petroleum-contaminated soil	Y	
WP_338601566.1	39.77	96	*Desulfoferula mesophila*	Lake sediment	Y	
WP_240081810.1	39.45	97	*Pelotomaculum*	UNK	UNK	
WP_277441990.1	39.43	97	*Pelotomaculum isophthalicicum*	Wastewater	Y	Phthalate
WP_006523843.1	39.39	95	*Desulfoscipio gibsoniae*	Freshwater mud	Y	
WP_006523931.1	39.39	95	*Desulfoscipio gibsoniae*	Freshwater mud	Y	
WP_028321792.1	39.39	96	*Desulfatiglans anilini*	Marine sediment	Y	4-chlorophenol
WP_066667006.1	39.31	95	*Desulfotomaculum copahuensis*	Geothermal system	Y	
WP_240984707.1	39.19	96	*Acididesulfobacillus acetoxydans*	Sediment	Y	
WP_041975195.1	39.08	95	*Pyrinomonas methylaliphatogenes*	Geothermal soils	N	
WP_073030992.1	38.93	97	*Desulfosporosinus*	UNK	UNK	
WP_224958034.1	38.91	95	*Geomonas fuzhouensis*	Paddy soil	Y	
WP_239027868.1	38.91	95	*Geomonas subterranea*	Paddy soil	Y	
WP_185243023.1	38.91	95	*Citrifermentans bremense*	Paddy soil	Y	
WP_302048708.1	38.89	98	*Desulfosporosinus nitroreducens*	Subsurface sediment	Y	
WP_028894729.1	38.87	95	*Syntrophorhabdus aromaticivorans*	Anaerobic granular sludge	Y	Phenol
WP_145025204.1	38.86	95	*Geobacter argillaceus*	Clay mineral	Y	
WP_044346694.1	38.82	96	*Dethiosulfatarculus sandiegensis*	Marine sediment	Y	Paraffin

Table S2. Bacterial strains used in this work.

Strains	Description / Use	Source
**NEB 10-beta competent *Escherichia coli***	Cloning strain	NEB
Escherichia coli BL21(DE3)	Protein expression and purification	
Escherichia coli TP1000	Protein expression and purification	Dr. Kurt Warnhoff
Gordonibacter sp. 28C	Metabolism assay, RNA-seq	Dr. Peter Turnbaugh
Paraeggerthella hongkongensis RC2/2 A	Metabolism assay	
Eggerthella lenta A2	Metabolism assay	
Eggerthella sinensis DSM 16107	Metabolism assay	
Gordonibacter urolithinfaciens DSM 27213	Metabolism assay, protein expression and purification	DSMZ
Gordonibacter pamelaeae 7-10-1b	Metabolism assay	
Raoultibacter massiliensis DSM 103407	Metabolism assay	
Adlercreutzia equolifaciens DSM 19450	Metabolism assay	
Senegalimassilia anaerobia JC 110	Metabolism assay	
Ellagibacter isourolithinifaciens DSM 104140	Metabolism assay	
Rubneribacter badeniensis DSM 103407	Metabolism assay	
Enterocloster bolteae DSM 15670	Metabolism assay, RNA-seq	
Enterocloster asparagiformis DSM 15981	Metabolism assay	

Table S3. Plasmids used in this work.

Plasmids	Description	Source
pMB-pET28a-Eah	Expression vector for Eah in *E. coli* BL21(DE3)	This work Addgene ID: 249564
pMB-pXD80-Eadh1	Expression vector for Eadh1 in *G. urolithinfaciens* DSM 27213	This work Addgene ID: 249567
pMB-pXD80-Eadh2	Expression vector for Eadh2 in *G. urolithinfaciens* DSM 27213	This work Addgene ID: 249568
pMB-pXD80-Eadh3	Expression vector for Eadh3 in *G. urolithinfaciens* DSM 27213	This work Addgene ID: 249569
pTrcHis 2A	Background expression vector for Ucdh expression in *E. coli* TP1000	ThermoFisher
pMB-pTrcHis2A-Ucdh	Expression vector for Ucdh in *E. coli* TP1000	This work Addgene ID: 249565
pMB-pTrcHIs2A-Ucdh-XdhC	Expression vector for Ucdh with XdhC in *E. coli* TP1000	This work Addgene ID: 249566

Table S4. Oligonucleotides used in this work.

Oligonucleotides	Sequence (5'→3')
oMB-Eah-F	AGCAGCCATCATCATCATCATCACAGCAGCGCGGAAAACAAGGTTATCGATAT CA
oMB-Eah-R	ATCTCAGTGGTGGTGGTGGTGGTGCTCGAGTTACAGGTTGAACAGCTTCGC
oMB-pET28a-F	CTCGAGCACCACCACCAC
oMB-pET28a-R	GCTGCTGTGATGATGATGATGATG
oMB-Eadh1-F	ACGAGGAGAGGAAAGAGGATTTAAAATGCATCACCATCACCATCAC
oMB-Eadh1-R	CCGAAGGTGAGCCAGTGTGAGTCGATTACCGGTAGATCGGTGTAACT
oMB-Eadh2-F	ACGAGGAGAGGAAAGAGGATTTAAAATGCATCACCATCACCATCAC
oMB-Eadh2-R	CCGAAGGTGAGCCAGTGTGAGTCGACTAGCCCTTCTTCGCTGGC
oMB-Eadh3-F	ACGAGGAGAGGAAAGAGGATTTAAAATGCATCACCATCACCATCAC
oMB-Eadh3-R	CCGAAGGTGAGCCAGTGTGAGTCGACTAGCTTGCGGGAATCTGCC
oMB-Ucdh-F	ATTAAATAAGGAGGAATAAACCATGGTATTACCTCAATTCGAGTATCTTGCT
oMB-Ucdh-R	TGAGATGAGTTTTTGTTCGGGCCCATTATTTTGAATTCCTGTCATCCTTCA
oMB-XdhC-F	GACTACGACATCCCGACTACCATGAAAACATTATTTACAGAGTTAAGAC
oMB-XdhC-R	TGAGATGAGTTTTTGTTCGGGCCCATTATTTCTTTTTACCCATCCTTTCAGCT
oMB-Ucdh-His-F	ACCATCACCATCACGACTACGACATCCCGACTACCGACTGTGACAAACATTATT TTAAAAAACCG
oMB-Ucdh-His-R	CGTAGTCGTGATGGTGATGGTGATGTTTATGCATTCTTTGCTTCCTCCTC
oMB-Ucdh-R	GGTAGTCGGGATGTCGTAGTCTTATTTTGAATTCCTGTCATCCTTCATG
oMB-Ucdh-F	ATTAAATAAGGAGGAATAAACCATGGTATTACCTCAATTCGAGTATCTTGCT
oMB-Ucdh-noXdhC-R	TGAGATGAGTTTTTGTTCGGGCCCATTATTTTGAATTCCTGTCATCCTTCA

Table S5. Gene locus for enzymes identified in this work.

Protein name	Source organisms	Gene locus	Protein ID
Eah	*Gordonibacter sp.* 28C	C1878_RS16320	WP_114602621.1
Eadh1 (catalytic subunit)	*Gordonibacter sp.* 28C	C1878_RS16305	WP_114602618.1
Eadh2 (catalytic subunit)	*Gordonibacter sp.* 28C	C1878_RS11985	WP_114601864.1
Eadh3 (catalytic subunit)	*Ellagibacter isourolithinifaciens* DSM 104140	F8C90_RS00245	WP_158048609.1
Ucdh (catalytic subunit)	*Enterocloster bolteae* DSM 15670	CGC65_RS20420	WP_002569573.1
XdhC	*Enterocloster bolteae* DSM 15670	CGC65_RS20280	WP_002565223.1

SI References

1. A. A. Shishkin, *et al.*, Simultaneous generation of many RNA-seq libraries in a single reaction. *Nat. Methods* **12**, 323–325 (2015).

2. T. Paysan-Lafosse, *et al.*, InterPro in 2022. *Nucleic Acids Res.* **51**, D418–D427 (2023).

3. N. Jha, *et al.*, Gaia: An AI-enabled genomic context–aware platform for protein sequence annotation. *Sci. Adv.* **11**, eadv5109 (2025).

4. B. Langmead, S. L. Salzberg, Fast gapped-read alignment with Bowtie 2. *Nat. Methods* **9**, 357–359 (2012).

5. G. H. Putri, S. Anders, P. T. Pyl, J. E. Pimanda, F. Zanini, Analysing high-throughput sequencing data in Python with HTSeq 2.0. *Bioinformatics* **38**, 2943–2945 (2022).

6. M. I. Love, W. Huber, S. Anders, Moderated estimation of fold change and dispersion for RNA-seq data with DESeq2. *Genome Biol.* **15**, 550 (2014).

7. A. Zhu, J. G. Ibrahim, M. I. Love, Heavy-tailed prior distributions for sequence count data: removing the noise and preserving large differences. *Bioinformatics* **35**, 2084–2092 (2019).

8. M. E. Ritchie, *et al.*, limma powers differential expression analyses for RNA-sequencing and microarray studies. *Nucleic Acids Res.* **43**, e47 (2015).
